# Extracellular vesicles from KSHV-infected endothelial cells activate the complement system

**DOI:** 10.18632/oncotarget.21668

**Published:** 2017-10-09

**Authors:** Hyungtaek Jeon, Seung-Min Yoo, Hyo Sun Choi, Ji Young Mun, Hee-Gyoo Kang, Jiyeong Lee, Jinsung Park, Shou-Jiang Gao, Myung-Shin Lee

**Affiliations:** ^1^ Department of Microbiology and Immunology, Eulji University School of Medicine, Daejeon, South Korea; ^2^ BK21 Plus Program, Department of Senior Healthcare, Graduate School, Eulji University, Daejeon, South Korea; ^3^ Department of Biomedical Laboratory Science, College of Health Sciences, Eulji University, Gyeonggi-Do, Seongnam, South Korea; ^4^ Department of Urology, Eulji University School of Medicine, Daejeon, South Korea; ^5^ Department of Molecular Microbiology and Immunology, Keck School of Medicine, University of Southern California, Los Angeles, CA, USA

**Keywords:** extracellular vesicles, KSHV, complement system, alternative complement pathway, endothelial cells

## Abstract

Extracellular vesicles (EVs), released by cells, are associated with cell-to-cell communication and regulate various cellular processes. EVs draw parallels with viruses for their similar structures and functions. Increasing evidences from recent studies indicate that cells infected with viruses release a variety of EVs. Delineating the functions and mechanisms of EVs released during virus infection is essential for understanding the molecular basis of viral infection and replication as well as associated pathogenesis. The most challenging obstacle for these studies is the separation of EVs from viruses. In this study, we successfully isolated the EVs from *de novo* Kaposi’s sarcoma-associated herpesvirus (KSHV) infected-human endothelial cells during the period between virus entry and production. Intriguingly, a proteomics analysis of these EVs has revealed alterations of the complement system. Additionally, we have discovered that the EVs from KSHV-infected endothelial cells are potent activators of an alternative pathway of the complement system via exploitation of the endogenous C3 complement protein and properdin. Furthermore, we have found that complement activation promotes KSHV persistent latent infection by activating the NF-κB pathway, which enhances the survival of KSHV-infected cells and inhibits viral lytic replication. Our work identifies a novel role of EVs induced by KSHV during *de novo* infection and the underlying mechanism of complement activation by EVs.

## INTRODUCTION

Extracellular vesicles (EVs) are membrane-enclosed vesicles released by cells and play a role in cell-to-cell communication during various physiological and pathological processes, including inflammation and cancer [1, 2]. EVs contain a high concentration of RNAs, proteins, DNA, microRNAs, and bioactive lipids. Although EVs can be taken up by the surrounding cells to regulate multiple biological processes, their precise roles are still not fully understood [3]. EVs in host-pathogen interactions have been highlighted in several studies [4]. Specifically, viruses share the cellular vesiculation machinery with EVs [5], both of which have similar lipid composition and protein content. Because of their common biogenesis paths, viruses and EVs may be close relatives and the production of EVs could be altered during virus infection and production. Increasing evidences suggest that EVs are linked to viral pathogenesis. For instance, exosomes from human immunodeficiency virus (HIV)-infected cells enhanced the susceptibility of cells to infection and the exosomes from HIV-positive patients were significantly enriched with many cytokines [6, 7].

Kaposi’s sarcoma-associated herpesvirus (KSHV) is the etiologic agent of Kaposi’s sarcoma (KS), which is the most common cancer in acquired immune deficiency syndrome (AIDS) patients [8]. KS is composed of vascularizing spindle endothelial cells and infiltrating inflammatory cells [9]. KSHV encodes various immune modulatory viral proteins [10].

To study the EVs in viral infection, it is necessary to first separate EVs and virions. However, in some viral infections, it is almost impossible as both EVs and viruses have comparable size and buoyant density. Gammaherpesviruses, including KSHV, have latent and lytic replication phases, and hence, EVs can be isolated without contamination of virions during the latent phase of infection. Several studies have shown interesting results with the EVs isolated from latent phase-virus infected cells [11–13]. However, their exact role and mechanism remain mystified. Furthermore, EVs from *de novo* infected cells have not been investigated because of the difficulty in separation of EVs from virions. In this study, we have isolated EVs from *de novo* KSHV-infected human endothelial cells during the period between viral entry and virion production. Proteomics analysis of EVs from KSHV-infected cells showed an association with the complement system. We have found that these EVs potently activate the alternative complement pathway by exploiting the endogenous C3 and properdin. Finally, we have shown that complement activation confers a survival benefit to KSHV-infected human endothelial cells by activating the NF-kB and inhibiting viral lytic replication. Taken together, these findings reveal a novel mechanism by which KSHV manipulates the host innate immunity through the EVs pathway, thereby providing new insights into the pathogenesis of KSHV.

## RESULTS

### Isolation of EVs from de novo KSHV-infected primary human endothelial cells

**Figure 1:**
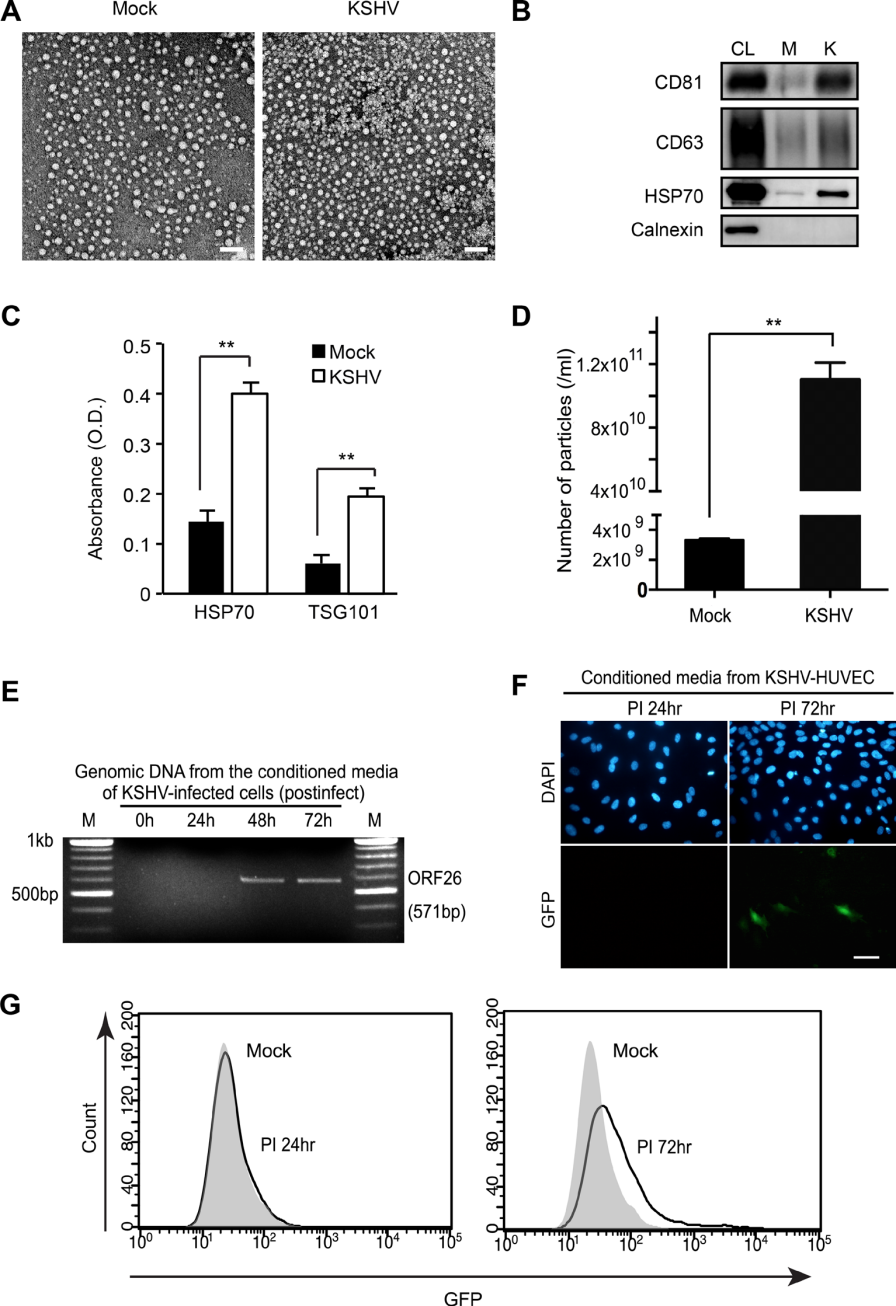
Isolation of extracellular vesicles (EVs) from KSHV-infected primary human endothelial cells (**A**) Electron microscopic images of EVs isolated from supernatants of mock- or KSHV-infected human umbilical vein endothelial cells (HUVECs) at 24 hpi. Scale bar: 100 nm. (**B**) Western blotting for EVs markers in EVs from mock- (M) or KSHV-infected HUVECs (K). CL: cell lysate. (**C**) Detection of HSP70 in EVs isolated from supernatants of mock- or KSHV-infected HUVECs by Enzyme linked immunosorbent assay (ELISA). Results are shown as mean ± SD, N=3, ^**^p < 0.01. (**D**) Microparticle number analysis of EV preparation from mock- and KSHV-infected HUVECs at 24 hpi. Microparticle number was analyzed by nanoparticle tracking analyzer, ZetaView. Results are shown as mean ± SD, *N* = 5, ^**^p < 0.01. (**E**) Detection of KSHV virion DNA by PCR. To detect KSHV DNA, virions were isolated from the supernatants of KSHV-infected HUVECs at 0, 24, 48, and 72 hpi by ultracentrifugation. The pellet was treated with RNase-free DNase I, followed by genomic DNA extraction. Then, KSHV ORF26 region was amplified by PCR. (**F**–**G**) Infectious KSHV is absent in supernatants of KSHV-infected HUVECs at 24 hpi. Supernatants were collected at 24 hpi and 72 hpi, concentrated 30X, and used to infect naïve HUVECs. After infection, green fluorescence protein (GFP) expression was analyzed by fluorescence microscopy or flow cytometry to monitor infection. Nuclei were stained with 4′,6-diamidino-2-phenylindole (DAPI). Scale bar: 50 μm.

It was known from previous studies that KSHV virions are not produced before 24 hours of post-infection (hpi) during primary KSHV infection of human primary umbilical vein endothelial cells (HUVECs) [14, 15]. We have developed procedures to isolate EVs in the supernatant of culture of *de novo* KSHV-infected HUVECs without the contamination of KSHV virions. At 1 hpi, the cells were extensively washed with PBS to eliminate the virus inoculum and supplemented with fresh culture media. The infected cells were then cultured for 24 hours, and the supernatant was collected for EVs isolation. Electron microscopy revealed that most of the isolated EVs were around 30–40 nm, which were much smaller than KSHV particles, and were free of KSHV particles (Figure 1A). The isolated EVs were verified for the presence of known EV markers by Western-blotting (Figure 1B) and ELISA (Figure 1C) [16, 17]. HSP70 is a membrane protein of exosome and can be detected by ELISA [17, 18]. There were significantly higher levels of HSP70 in EVs from the supernatant of KSHV-infected HUVECs (KSHV-HUVECs) than mock-infected HUVECs (mock-HUVECs) at 24 hpi. In nanoparticle tracking analysis with ZetaView, the number of particles detected from KSHV-HUVECs was about 30-fold higher than that from mock-HUVECs (Figure 1D). The existence of virions in the isolated EVs was analyzed by PCR and fluorescent microscopy. As expected, KSHV genome was not detected in the EVs from KSHV-HUVECs at 24 hpi (Figure 1E). We used a recombinant KSHV BAC16, which expresses a green fluorescence protein (GFP) cassette [19], to monitor the infection. We did not observe any GFP-positive cells in culture inoculated with supernatant from KSHV-HUVECs at 24 hpi (Figure 1F), thus confirming the lack of production of infectious virions at this time point. To summarize, our results indicated that EVs were successfully isolated from the supernatant of *de novo* KSHV-infected human endothelial cells without any contamination of virions.

### Proteomic profile of EVs from mock- and KSHV-infected HUVECs

**Figure 2:**
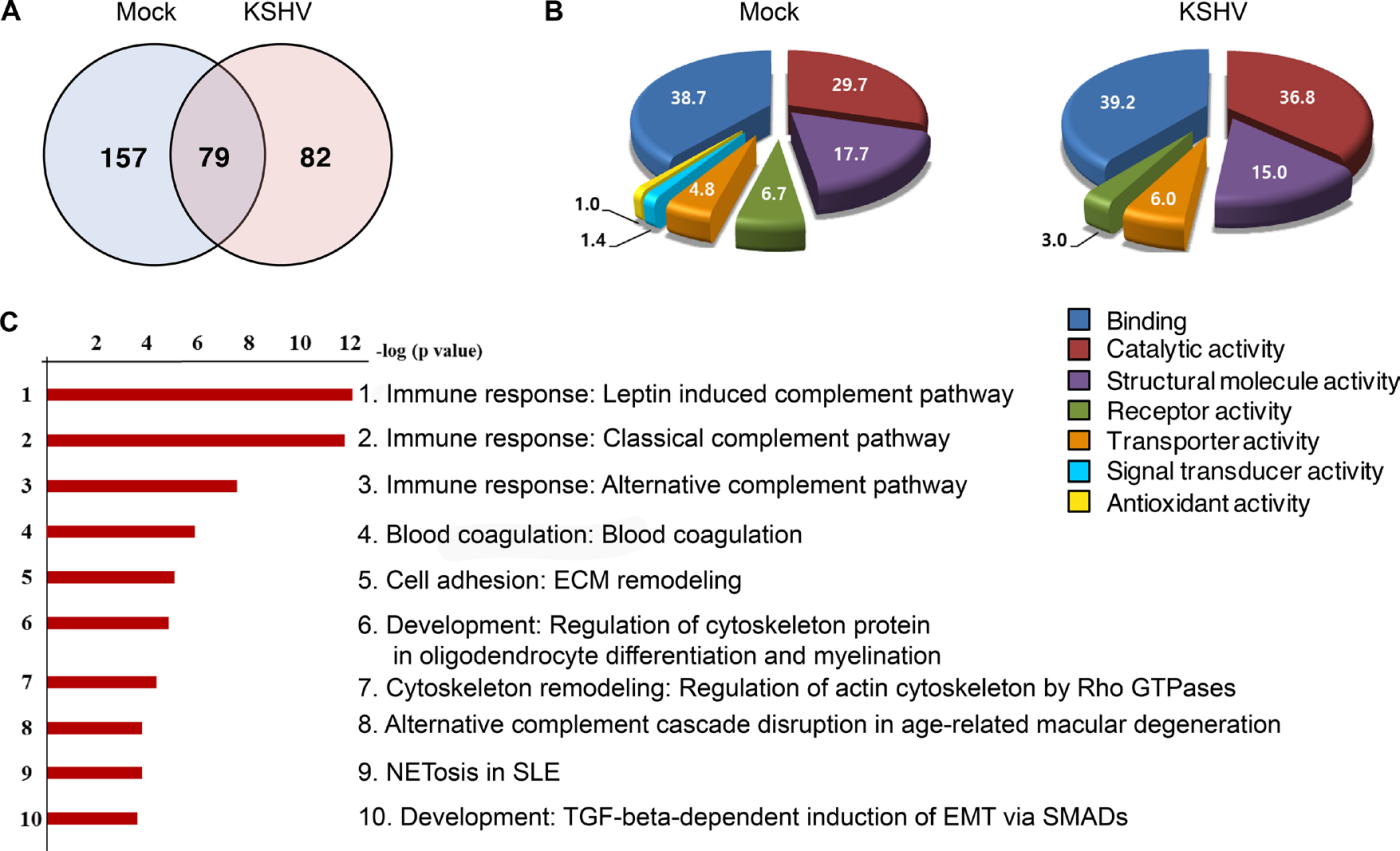
Proteome profile of EVs from mock- and KSHV-infected cells (**A**) Venn diagram depicts the overlap in protein profiles between EVs from mock- and KSHV-infected HUVECs. Seventy-nine proteins are co-expressed in both the groups, whereas, 157 and 82 proteins were unique to mock-infected (Mock) and KSHV-infected (KSHV) group, respectively. (**B**) The difference in the molecular functions of proteins isolated from mock and KSHV EVs are shown in the pie charts. The numbers indicate the percentages of proteins in relative to total protein. (**C**) Pathway map analysis of differentially expressed proteins between the mock and KSHV groups. The list is arranged in descending order with the most significant pathway at the top. Bar shows -log *p*-values.

Proteomics analysis was performed on EVs from supernatants of mock- and KSHV-HUVECs at 24 hpi (Figure 2 and Supplementary Table 1). KSHV infection altered the protein profile of EVs (Figure 2A). A total 318 proteins were identified by LC-MS/MS analysis, of which 239 proteins were found to be altered more than 2-fold following KSHV infection, which could be annotated to numerous molecular functions (Figure 2B). Proteins with signal transducer activity or antioxidant activity were detected in EVs from mock-infected cells though the levels were low but it was no longer detectable following KSHV infection. The pathway mapping of differentially expressed proteins was carried out using the GeneGo MetaCore software and the top hits were presented in decreasing statistical significance (Figure 2C). Ranked pathway lists were created based on the calculated *p*-values using hypergeometric test. The *p*-values that are calculated in Metacore were represented in the lowest order. The ranking showed the probabilities of pathways of the mapped proteins from our data compared to the pathways of proteins annotated with the GeneGo software. Among the top 10 pathways retrieved, the top 3 were related to complement activation of the immune response.

### Complement activation in primary endothelial cells during de novo KSHV infection

**Figure 3:**
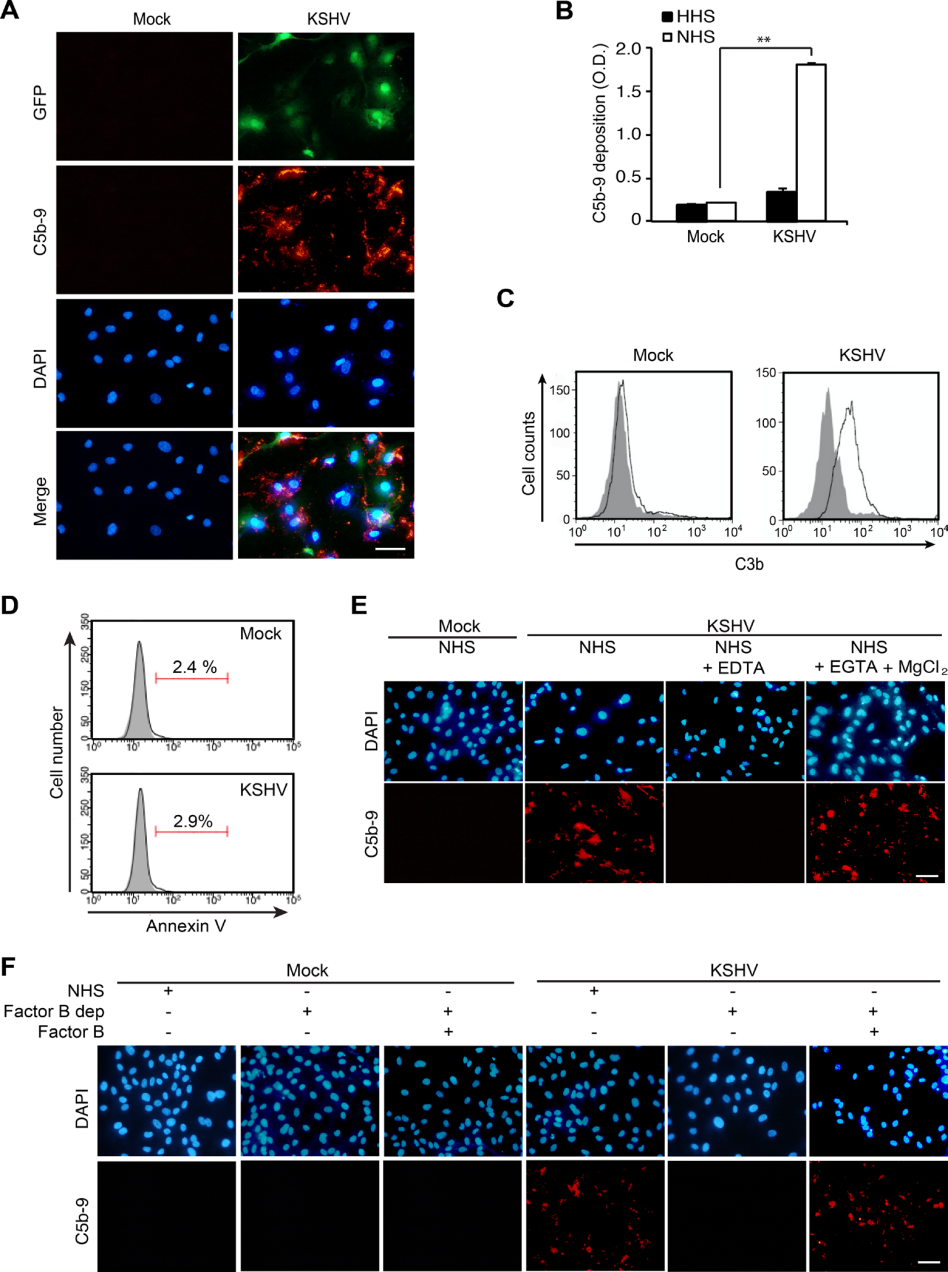
KSHV activates the alternative complement pathway during acute infection of primary human endothelial cells (**A**) Detection of C5b-9 depositions on HUVECs infected by KSHV. HUVECs were infected with KSHV for 24 h, treated with normal human serum (NHS) for another 30 min, and examined for C5b-9 depositions by an immunofluorescence assay (IFA). Nuclei were stained with DAPI. Mock: mock-infected HUVECs, KSHV: KSHV-infected HUVECs, GFP: green fluorescent protein, C5b-9: C5b-9 deposition detected with an anti-C5b-9 antibody. Scale bar: 50 μm. (**B**) Detection of C5b-9 depositions on mock- or KSHV-infected HUVECs treated with heat-inactivated human serum (HHS) or NHS using a cell-based enzyme linked immunosorbent assay (ELISA). Results are shown as mean ± SD, *N* = 3, ^**^p < 0.01. (**C**) Analysis of C3b surface deposition levels by flow cytometry. NHS-treated mock- or KSHV-infected HUVECs were stained with an anti-C3b antibody. Gray and white areas represent the isotype control and anti-C3b antibody, respectively. (**D**) KSHV does not induce apoptosis during the early phases of acute infection in HUVECs. KSHV-infected HUVECs at 24 hpi were examined for Annexin V-positive cells by flow cytometry analysis. Representative figures from the Annexin V assay are shown. (**E**) EDTA, but not EGTA and MgCl2, abolish complement activation during de novo KSHV infection of HUVECs. Mock- or KSHV-infected cells incubated with NHS or NHS containing either 20 mM EDTA or 10 mM EGTA with 20 mM MgCl2 were examined for C5b-9 depositions by IFA. Scale bar: 50 μm. (**F**) Factor B is required for complement activation in KSHV-infected HUVECs. Mock- or KSHV-infected HUVECs at 24 hpi treated with NHS, factor B-depleted human serum (factor B dep), or factor B-depleted human serum compensated with factor B were examined for C5b-9 depositions by IFA. Scale bar: 50 μm.

Our result from proteomics analysis of EVs indicated an alteration of the complement system. To investigate whether *de novo* KSHV infection would cause complement activation, we examined the deposition of membrane attack complex (MAC) or C5b-9 on mock- and KSHV-infected HUVECs at 24 hpi following incubation of the cells with normal human serum (NHS). The deposition of MAC was observed on KSHV- but not mock-infected cells (Figure 3A). To quantify the deposition of C5b-9 on cells, a cell-based enzyme linked immunosorbent assay (ELISA) technique was applied [20]. There were about 9-fold higher C5b-9 depositions on KSHV- than mock-infected cells treated with NHS (Figure 3B). Heat-inactivated human serum (HHS) was used as a negative control and C5b-9 deposition was not observed on HHS-treated KSHV- and mock-infected cells. Since C3b is known to bind covalently to cell surfaces after complement activation [21], we examined the presence of C3b on cell surfaces by flow cytometry. Only KSHV- but not mock-infected HUVECs showed a significant increase in cell surface-bound C3b (Figure 3C). Several previous studies have indicated that apoptosis is an initiating factor of complement activation [22, 23]. Hence, we examined apoptosis at 24 hpi by flow cytometry (Figure 3D). Both mock- and KSHV-infected HUVECs had very few apoptotic cells (< 3%), suggesting that complement activation observed during *de novo* KSHV infection was not due to apoptosis.

To identify the pathway of the complement system activated during KSHV infection, the infected cells were incubated with NHS containing 10 mM ethylenediaminetetraacetic acid (EDTA) or 10 mM ethyleneglycotetraacetic acid (EGTA) with 2 mM MgCl_2_. EDTA inhibits the activation of all the complement pathways while EGTA with 2 mM MgCl_2_ specifically inhibits the antibody-dependent classical complement pathway. EDTA-treated KSHV-infected cells no longer had C5b-9 deposition whereas cells treated with EGTA together with MgCl_2_ had C5b-9 deposition (Figure 3E). These results indicated that C5b-9 deposition on KSHV-infected cells was not mediated by the classical complement pathway. To determine whether the activation of alternative complement pathway was involved in the C5b-9 deposition during KSHV infection, we treated the cells with factor B depleted NHS. Factor B is an essential component for initiating the alternative complement pathway. C5b-9 cannot be assembled without factor B. As expected, depletion of factor B failed to induce C5b-9 deposition; however, the addition of factor B to the depleted human serum rescued the C5b-9 deposition (Figure 3F). The above results indicated that the alternative complement pathway was activated during *de novo* KSHV infection of HUVECs.

### KSHV activates the complement pathway in primary human endothelial cells during entry and trafficking stages of infection

**Figure 4:**
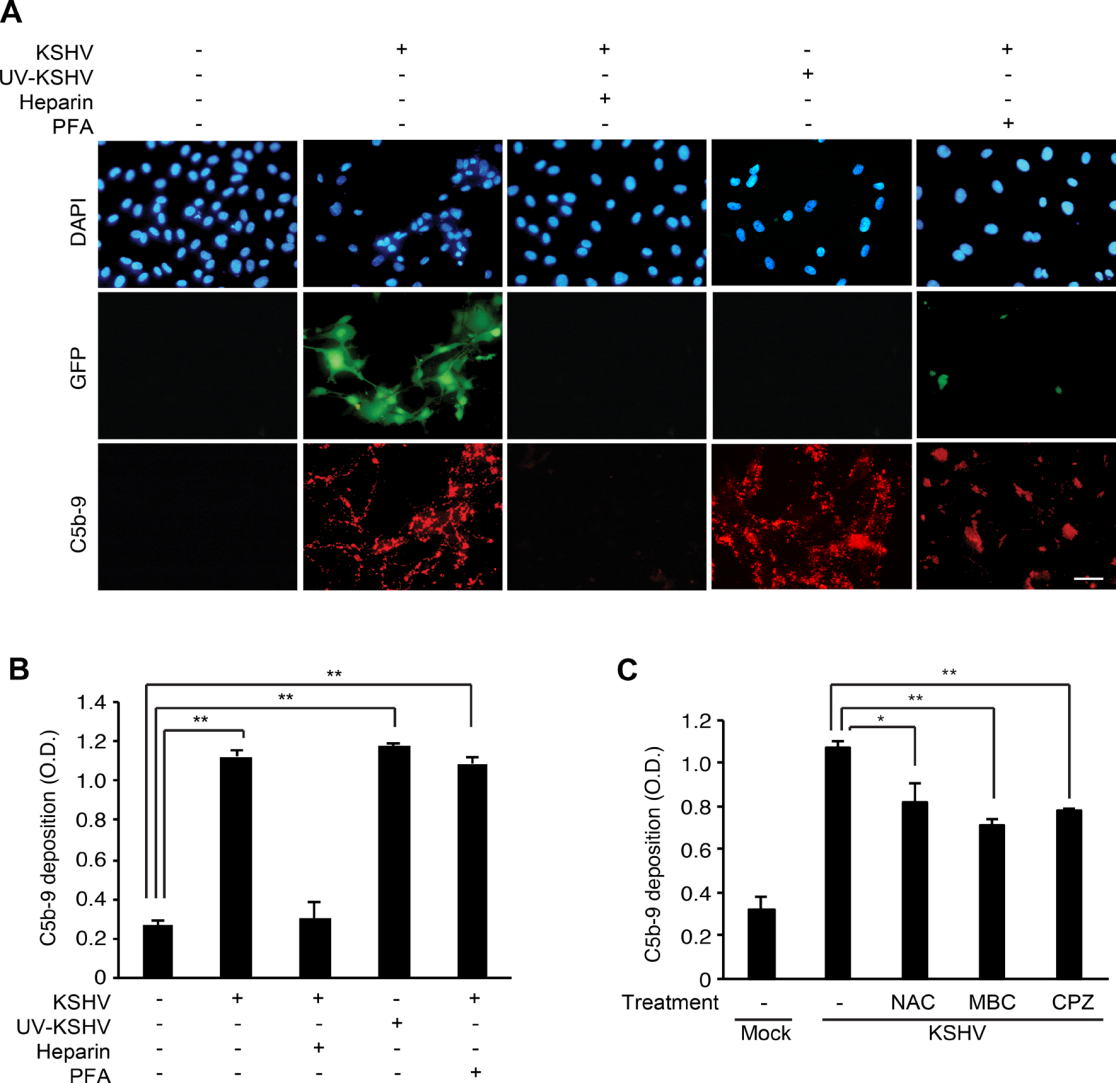
Complement activation depends on the entry and trafficking stages of KSHV infection (**A**–**B**) Effects of inhibiting early phases of KSHV infection on complement activation. HUVECs were treated with soluble heparin or phosphonoformic acid (PFA) before and during KSHV infection, or infected with UV-irradiated KSHV, and examined for surface deposition of C5b-9 by IFA (A) and a cell-based ELISA (B). Nuclei were stained with DAPI. Heparin: treatment with 10 μg/mL of soluble heparin, PFA: treatment with 300 μg/mL of phosphonoformic acid. Scale bar: 50 μm. OD: optical density. Results are shown as mean ± SD, *N* = 3, ^**^p < 0.01. (**C**) Effects of inhibitors preventing the entry of KSHV on complement activation. HUVECs were treated with a scavenger of reactive oxygen species N-acetyl-cysteine (NAC), an inhibitor of lipid raft formation methyl-β-cyclodextrin (MBC), or an inhibitor of clathrin-mediated endocytosis chlorpromazine (CPZ) for 1 h, infected with KSHV in the presence of the respective inhibitors for 24 h, and examined for C5b-9 depositions by a cell-based ELISA. Mock: mock-infected HUVECs, KSHV: KSHV-infected HUVECs, Results are shown as mean ± SD, *N* = 3, ^*^p < 0.05, ^**^p < 0.01.

To identify the stages of KSHV infection involved in activation of the complement pathway, we first examined the effect of blocking KSHV entry on complement activation by adding soluble heparin to the cells before or during KSHV infection. Soluble heparin blocks the binding of KSHV to heparin sulfate on cell surfaces, thereby inhibiting KSHV entry and infection [24]. Addition of soluble heparin inhibited C5b-9 deposition on the cell surface (Figure 4A and 4B), indicating that KSHV entry is essential for activating the complement pathway. Next, we exposed the virions to ultraviolet (UV) irradiation to cross-link the viral DNA, thus inactivating the virus and preventing the expression of viral genes. UV irradiation did not affect C5b-9 deposition (Figure 4A and 4B) indicating that the expression of KSHV genes was not required for activating the complement pathway. As expected, UV irradiation effectively abolished KSHV infection as indicated by the absence of GFP-positive cells (Figure 4A); however, it did not affect the entry and trafficking of KSHV particles as shown by the detection of ORF65-positive viral particles at the peripheral nuclear regions at 4 hpi (Supplementary Figure 1). Finally, we treated the infected cells with phosphonoformic acid (PFA) at 8 hpi to block the expression of KSHV late lytic genes. In agreement with the results of UV-irradiation, treatment with PFA did not affect C5b-9 deposition (Figure 4A and 4B), confirming that the expression of KSHV late lytic genes was not required for activating the complement pathway. These results indicated that KSHV activated the complement pathway during the early entry and trafficking phases of acute infection, which was independent of the expression of viral genes.

The early phase of KSHV infection consists of multiple stages, including attachment, viral glycoprotein-receptor interaction, entry mediated by endocytosis, cytoplasmic trafficking mediated by cytoskeleton dynamics, and delivery of KSHV DNA to the nucleus [25]. KSHV activates various signaling pathways and induces cytoskeleton remodeling and formation of lipid rafts during these stages of infection. For example, attachment of KSHV to the host cell activates FAK, Src and Rac1, and induces reactive oxygen species (ROS) [26]. To explore the relationship between the early stages of KSHV entry and complement activation, we treated the cells with an ROS inhibitor, N-acetyl-cysteine (NAC), to block viral entry [27] and examined C5b-9 deposition. Similarly, we also examined the effects of a lipid raft disruptive agent, methyl-β-cyclodextrin (MBC), and an inhibitor of clathrin-mediated endocytosis, chlorpromazine (CPZ), on complement activation [28]. All inhibitors for viral entry showed significant inhibitory effects on complement activation (Figure 4C), indicating that complement activation during KSHV infection was closely associated with the events of viral entry and trafficking.

To determine whether complement activation was specific to KSHV-infection of HUVECs, we examined complement activation during lentivirus infection of HUVECs (Supplementary Figure 2). In striking contrast to KSHV-infection of HUVECs, we did not detect any C5b-9 deposition on HUVECs infected by lentivirus at 24 hpi, indicating complement activation was not a general immune response to all virus infections in HUVECs but might be specific KSHV infection.

### EVs secreted by KSHV-infected endothelial cells activate the complement system

**Figure 5:**
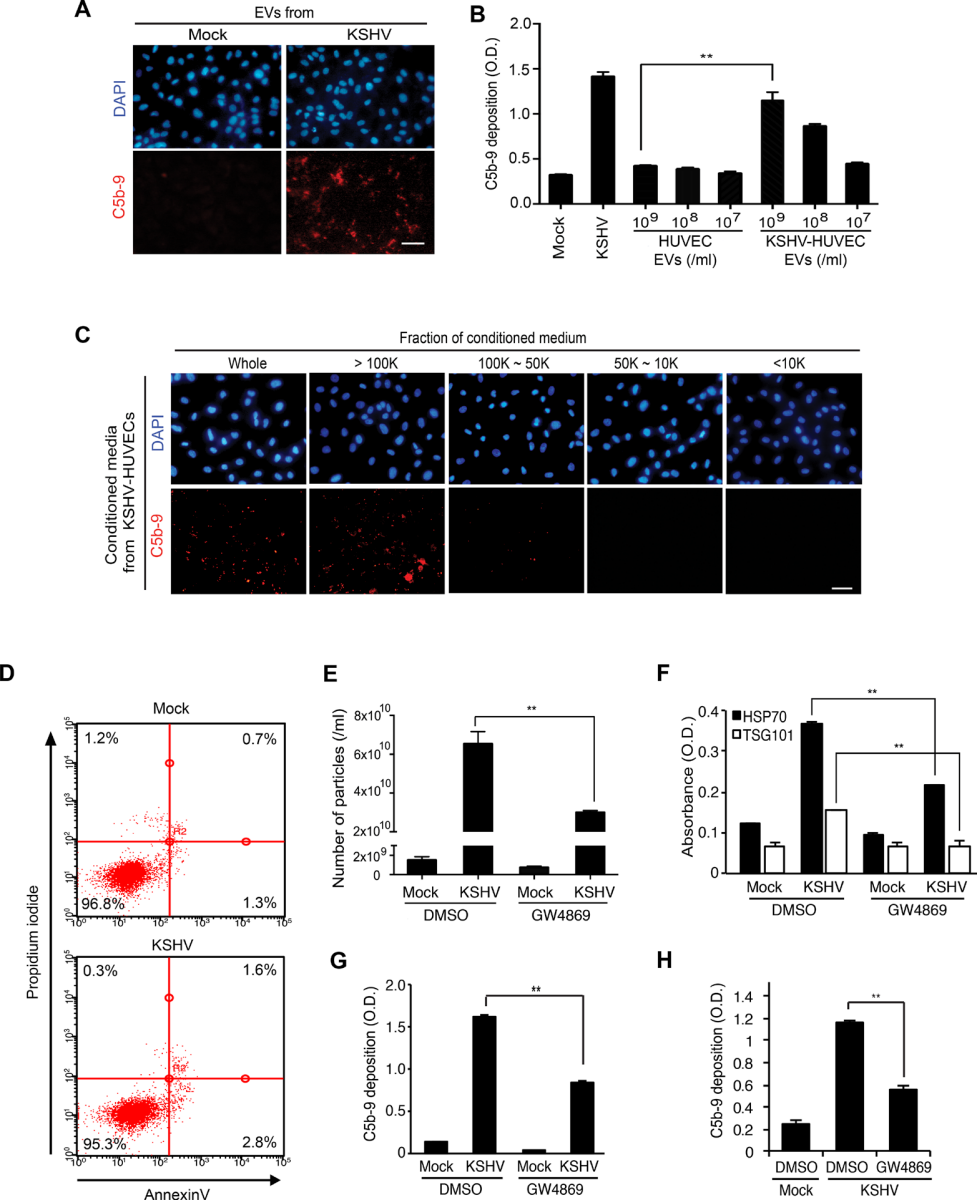
EVs from KSHV-infected human endothelial cells activate the complement system (**A**) EVs from KSHV-infected HUVECs activate the complement system. HUVECs treated with EVs from mock- or KSHV-infected HUVECs were examined for C5b-9 depositions. EVs isolated from the supernatant of mock- or KSHV-infected cells at 24 hpi were used to treat naive HUVECs for 24 h. After exposure to the EVs, all cells were treated with normal human serum (NHS) for another 30 min, and C5b-9 was analyzed by IFA. Scale bar: 50 μm. (**B**) Quantification of C5b-9 depositions on HUVECs treated with a same number of EVs from mock- or KSHV-infected HUVECs using a cell-based ELISA. Mock: mock-infected HUVECs, KSHV: KSHV-infected HUVECs. Results are shown as mean ± SD, *N* = 3, ^**^p < 0.01. (**C**) The high molecular weight fraction of supernatant from KSHV-infected cells induces complement activation. The supernatants were fractionated using Vivaspin 20 protein concentrator spin columns with molecular weight cut-offs of 100, 50, and 10 kDa. Each isolated fraction was used to treat uninfected HUVECs for 24 h, followed by treatment with NHS. C5b-9 depositions were analyzed by IFA. Scale bar: 50 μm. (**D**) EVs do not induce apoptosis and cell death. HUVECs treated with the EVs from mock- or KSHV-infected human endothelial cells were examined for apoptosis and dead cells by flow cytometry analysis following staining for Annexin V- and propidium iodide (PI)-positive cells. (**E**–**H**) Inhibition of EV biogenesis during de novo KSHV infection suppressed complement activation. HUVECs pretreated with dimethyl sulfoxide (DMSO) or 10 μM GW4869 for 1 h were either mock-infected (M) or infected with KSHV (K) in the presence of the respective agents. The supernatant (CM) was collected at 24 hpi and the inhibitory effect of GW4869 on the production of EVs was analyzed by counting microparticles number and examining the levels of HSP70 in the CM using nanoparticle tracking analyzer (E) and ELISA (F), respectively. Each CM was applied to naïve HUVECs for 24 h, followed by treating with NHS for 30 min. C5b-9 depositions were analyzed by cell-based ELISA (G) and IFA (Supplementary Figure 3A) (H) Suppression of complement activation in de novo KSHV-infected HUVECs by GW4869. HUVECs pretreated with GW4869 were infected by KSHV followed by exposing the cells with NHS. The depositions of C5b-9 were quantified by cell-based ELISA. Results are shown as mean ± SD, *N* = 3, ^**^p < 0.01.

Our next question was whether complement activation during KSHV acute infection was mediated by KSHV virions or EVs. Because KSHV-infected HUVECs did not produce any infectious virions at 24 hpi (Figure 1E and 1F) [14, 15], we examined whether EVs produced by KSHV-infected HUVECs at 24 hpi were sufficient to activate the complement system. We detected C5b-9 deposition on naïve HUVECs following incubation for 24 hours with EVs isolated from supernatants of mock- or KSHV-infected HUVECs at 24 hpi, and then with NHS for another 30 min (Figure 5A). Significantly, the EVs from KSHV- but not mock-infected cells induced C5b-9 deposition. Importantly, C5b-9 deposition induced by EVs from KSHV- but not mock-infected cells increased in a dose-dependent fashion (Figure 5B).

There are several different methods for isolating EVs [29]. The results so far were obtained using EVs isolated from the supernatants of cultured cells by differential ultracentrifugation. We then used EVs isolated with exoQuick-TC solution, which also showed induction of complement activation (data not shown). Additionally, we subjected supernatants from KSHV- and mock-infected HUVECs at 24 hpi to centrifugation-based molecular weight fractionation in membrane filters and isolated four fractions: >100 kDa, 100–50 kDa, 50–10 kDa, and < 10 kDa. Each of the fractions was used to treat freshly cultured naïve HUVECs for 24 h and C5b-9 deposition was analyzed by IFA (Figure 5C). We only observed C5b-9 deposition with the >100 kDa fraction from KSHV-infected HUVECs. These results confirmed that complement activation by EVs from KSHV-infected cells was not dependent on the isolation methods of EVs. Furthermore, we did not observe any increase in apoptosis and necrosis in naïve HUVECs following incubation with EVs of mock- and KSHV-infected cells, indicating that they were not involved with the observed complement activation (Figure 5D).

To further confirm that EVs secreted from KSHV-infected HUVECs activated the complement system, we inhibited the production of EVs with a common inhibitor of exosome secretion, GW4869 [30]. GW4869 inhibits neutral sphingomyelinase 2, which participates in exosome secretion by triggering the budding of exosomes into intracellular multi-vesicular bodies [31]. We detected the inhibitory effect of GW4869 on exosome secretion as shown in the reduced production of EVs enumerated as microparticles with ZetaView (Figure 5E) and reduced HSP70 detected by ELISA in the EV preparations (Figure 5F). Consistent with these results, the isolated EVs from KSHV-infected HUVECs treated with GW4869 induced significantly less C5b-9 deposition on naïve HUVECs (Figure 5G and Supplementary Figure 3A). Furthermore, addition of GW4869 to HUVECs before and during KSHV infection significantly suppressed C5b-9 deposition on KSHV-infected HUVECs (Figure 5H). Importantly, GW4869 did not compromise KSHV infection under these conditions (Supplementary Figure 3B). Together, these results indicated that the EVs secreted from KSHV-infected HUVECs induced complement activation during *de novo* KSHV infection.

### EVs-mediated complement activation is associated with the alteration of endogenous C3 and properdin

**Figure 6:**
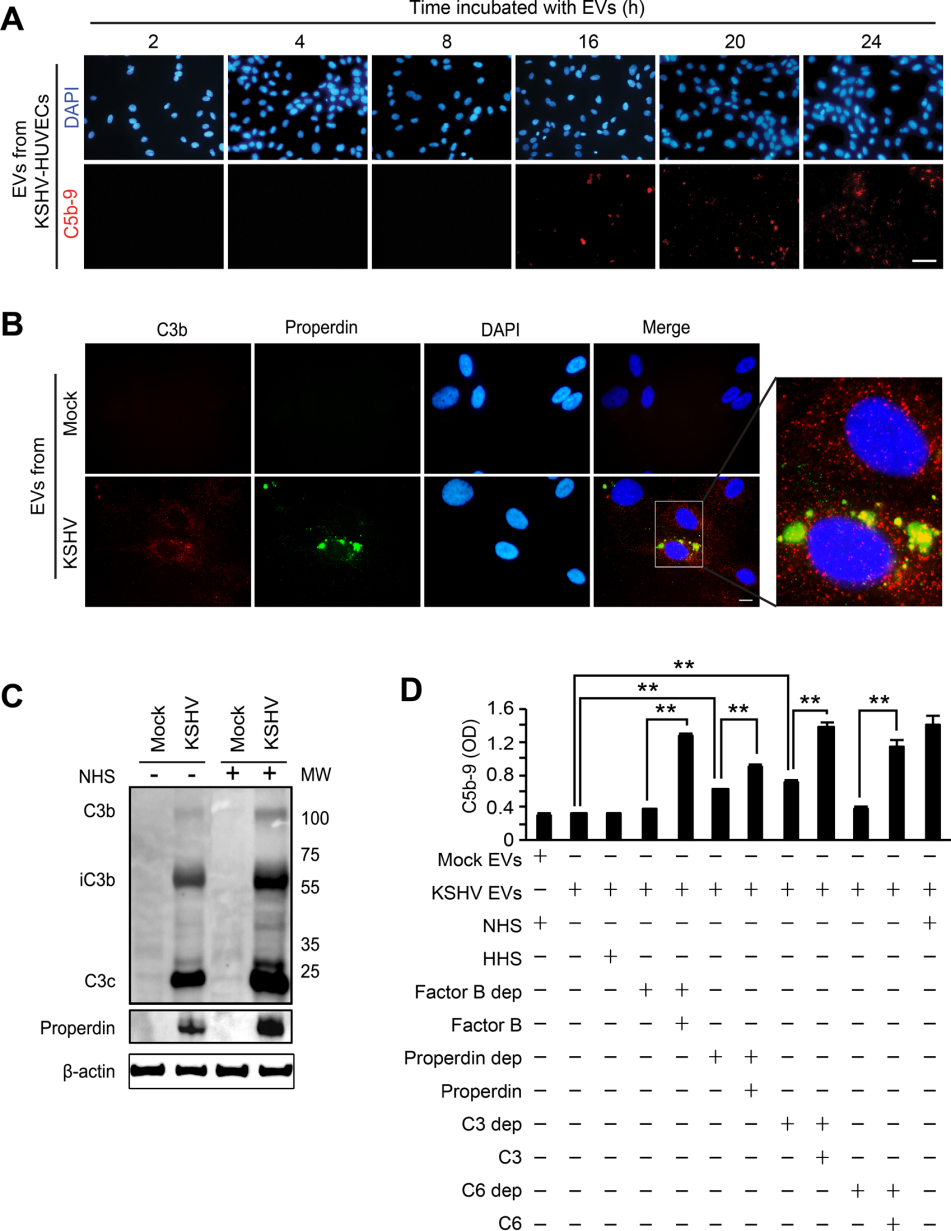
EVs-mediated complement activation is associated with the alteration of the endogenous C3 and properdin (**A**) EVs from KSHV-infected HUVECs are not a direct activator for the complement system. HUVECs treated with EVs from KSHV-infected HUVECs at 24 hpi for different lengths of time were examined for C5b-9 deposition. After exposure to the EVs for the indicated durations, the cells were treated with NHS for another 30 min, and analyzed for C5b-9 depositions by IFA. Scale bar: 50 μm. (**B**) Endogenous C3b and properdin were detected on the HUVECs treated with EVs from KSHV-infected cells. Naïve HUVECs were treated with EVs from mock- or KSHV-infected HUVECs for 24 h, followed by analyzing for C3b and properdin on the cell surface by IFA. Scale bar: 10 μm. (**C**) Western blotting for C3b and properdin. Naïve HUVECs were treated with EVs from mock- or KSHV-infected HUVECs, and cell lysates were prepared from these cells with or without treatment with NHS for 30 min. β-actin was used as a loading control. Mock: cell lysate of HUVECs treated with EVs from mock-infected cells, KSHV: cell lysate of HUVECs treated with EVs from KSHV-infected cells. (**D**) Cell ELISA for the depositions of C5b-9 with single complement factor-depleted human serum. Naïve HUVECs were treated with EVs from mock- (Mock Evs) or KSHV-infected cells (KSHV EVs) followed by exposure for 30 min to human serum with depletion of the indicated individual complement factor with and without the addition of the respective purified complement protein. Then C5b-9 depositions were quantified by cell ELISA. Results are shown as mean ± SD, *N* = 5, ^**^p < 0.01.

The EVs from the supernatant of KSHV-infected HUVECs were collected at 24 hpi, applied to naïve HUVECs, and monitored for C5b-9 deposition over a 24 h period (Figure 6A). The C5b-9 deposition was not detected until 16 h after treatment with the EVs, suggesting that complement activation was not mediated by direct binding of complement activators present in the EVs, rather the EVs may modify endothelial cells to trigger the activation of the complement system over time. Multiple mechanisms regulate the inherent activation of the complement system. For instance, high levels of complement regulatory proteins such as CD46, CD55, and CD59 on cell surfaces inhibit complement activation [21]. In latent KSHV-infected endothelial cells, some complement regulatory proteins are downregulated, resulting in activation of the complement system [32]. We have investigated if a similar mechanism might mediate the complement system during *de novo* KSHV infection of endothelial cells. The level of CD46, CD55 or CD59 mRNA in KSHV-infected cells was either increased or remained unchanged within 48 hpi (Supplementary Figure 4A). The results of flow cytometry and Western-blotting confirmed that there was no downregulation of any complement regulatory proteins during acute KSHV infection (Supplementary Figure 4B and C), indicating that complement activation during *de novo* KSHV infection was not caused by suppression of these proteins.

**Figure 8:**
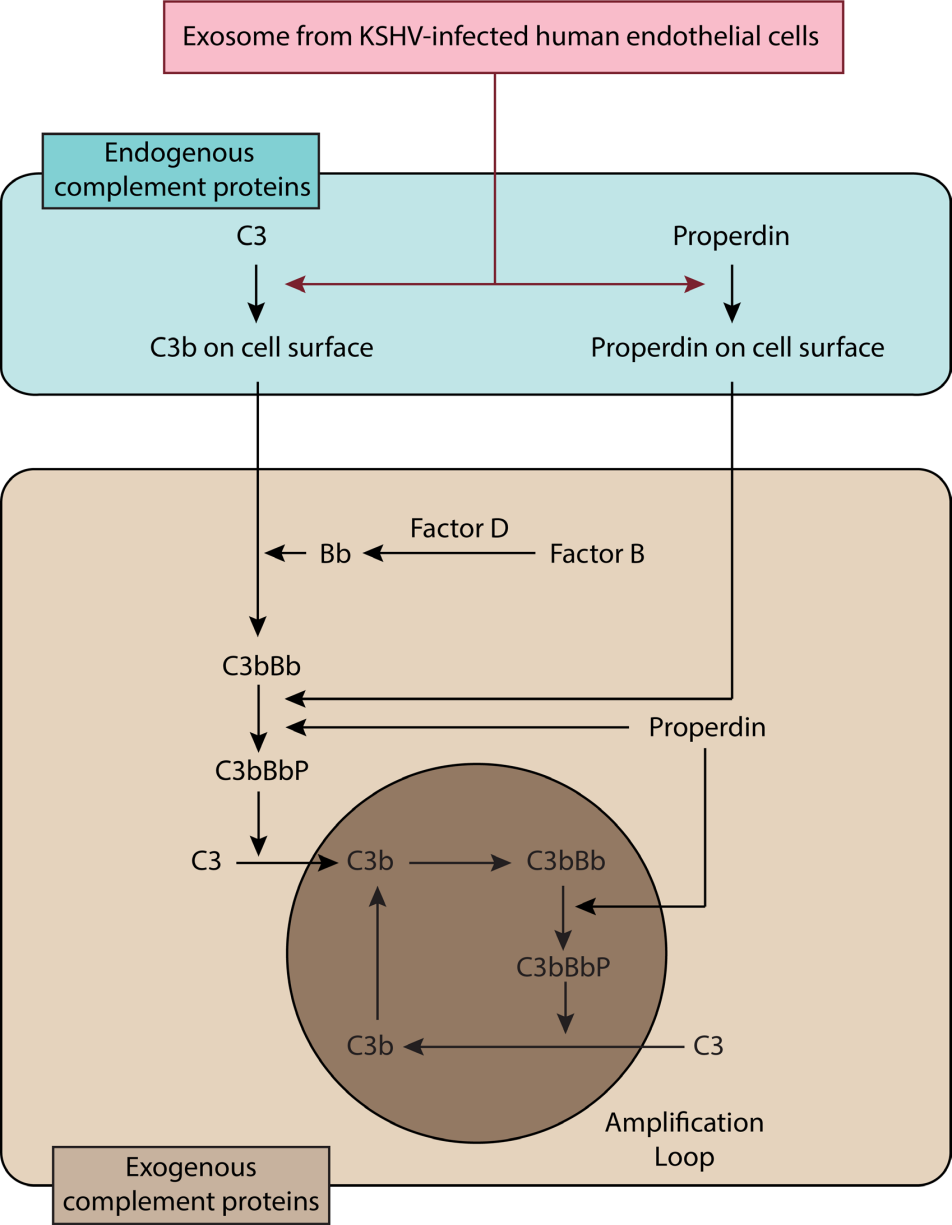
Schematic summary for the activation of complement system by EVs from de novo KSHV-infected primary human endothelial cells

In the alternative complement pathway, properdin is the only known positive regulator of complement activation. Properdin acts either as a stabilizer of C3bBb [33] or as an initiator of the pathway by binding independently of C3b to cell surfaces [34]. HUVECs are known to produce C3 and properdin [35, 36]. Thus, we examined C3b and properdin on HUVECs treated with the EVs from KSHV-infected HUVECs (Figure 6B). However, C3 and properdin are secreted proteins that are not present on the cell surface without complement activation. Surprisingly, we observed depositions of C3b and properdin on the surface of the EVs-treated HUVECs even when the cells were not treated with any human serum complements. EVs from the KSHV- but not mock-infected cells induced the depositions of C3b and properdin on HUVECs. Interestingly, C3b and properdin showed different staining patterns on the cell surface, suggesting that properdin might independently bind to cell surfaces. In agreement with the IFA results, strong iC3b, C3C and properdin, and some C3b were detected by Western-blotting in HUVECs treated with EVs from KSHV- but not mock-infected cells (Figure 6C). Although the addition of NHS could enhance the levels of C3b and properdin, we detected these complement proteins in EVs-treated cells without exogenous complement proteins (Figure 6C). As an indicator of complement activation [37], the detection of iC3b unequivocally indicated that endogenous C3 was activated by EVs isolated from KSHV-HUVECs and this activation process did not require the presence of exogenous complement proteins. In KSHV-infected HUVECs, C3b on the cell surface was detected, which is consistent with EV-mediated activation of C3. Additionally, properdin was also detected in KSHV-infected HUVECs at 24 hpi. (Supplementary Figure 5). To investigate the effect of endogenous C3 and properdin in the assembly of C5b-9 complex, HUVECs treated with EVs from mock- or KSHV-infected cells were incubated with human serum with individual complement factors depleted and the deposition of C5b-9 was quantified by cell-based ELISA (Figure 6D). The increased C5b-9 deposition was observed in the cells treated with properdin-depleted or C3-depleted serum but not factor B-depleted or C6-depleted serum, indicating that the endogenous properdin and C3 were sufficient to cause C5b-9 deposition and complement activation. As expected, addition of the respective complement protein including properdin and C3 protein increased C5b-9 deposition, indicating that further complete activation of the complement system required exogenous complement proteins (Figure 6D and Figure 8).

**Figure 7:**
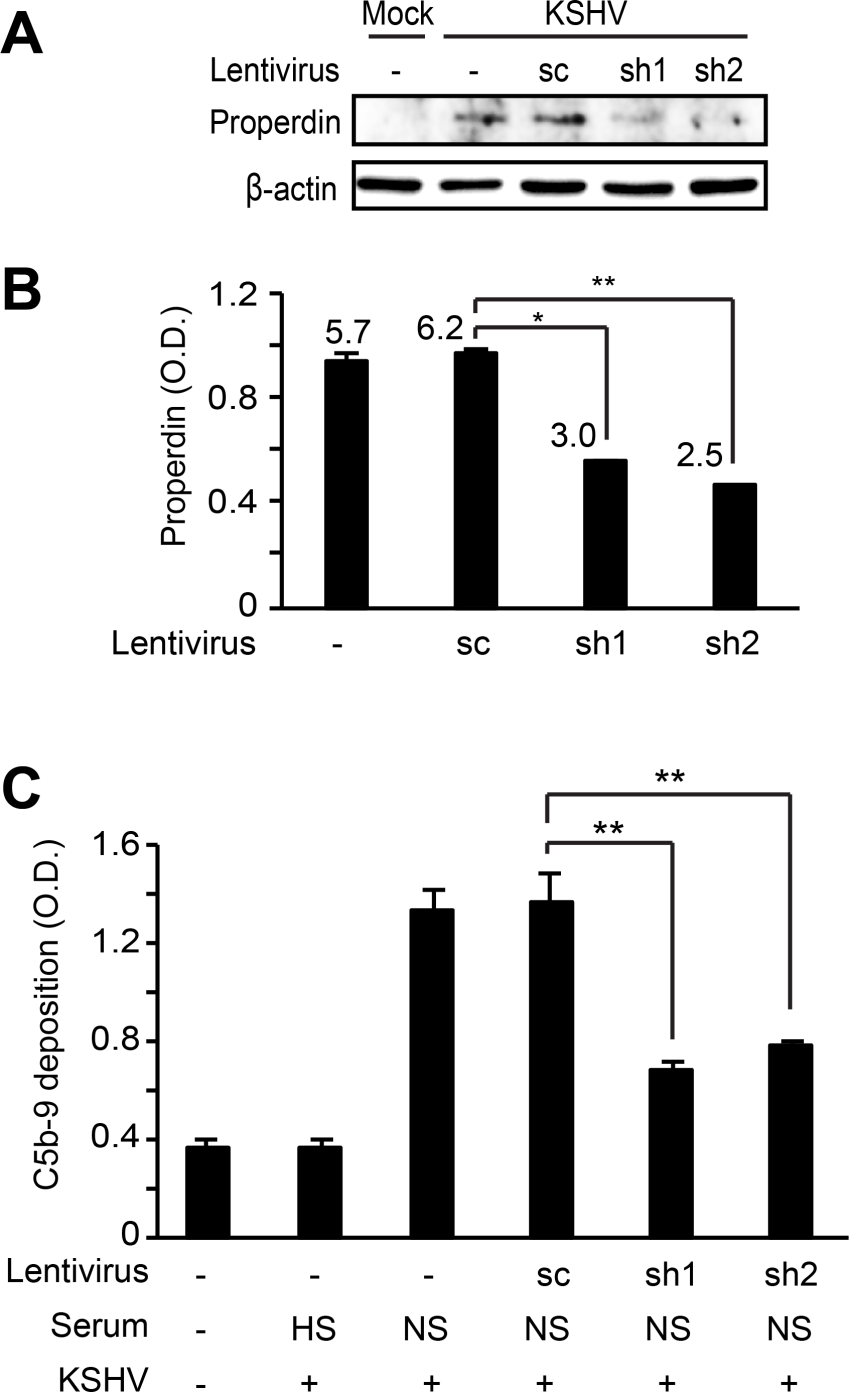
Knockdown of properdin in HUVECs suppresses complement activation by KSHV infection HUVECs transduced with lentiviruses containing properdin-specific shRNAs (sh1 and 2) and selected with puromycin for 10 days were infected with KSHV for 24 h. Properdin expression was examined by western blotting (**A**) and the secreted properdin in supernatants was quantified using a human properdin ELISA kit (**B**). Numbers represented on the graph indicate concentrations of properdin (ng/mL) after calibration with purified recombinant properdin. (**C**) Complement activation was examined by quantifying C5b-9 deposition using a cell-based ELISA. sc: scrambled shRNA, HS: heat-inactivated human serum, NS: normal human serum. Results are shown as mean ± SD, *N* = 3, ^*^p < 0.05, ^**^p < 0.01.

To further confirm that the endogenous properdin played a role in complement activation, we performed shRNA knock down of properdin and examined the effect on complement activation during acute KSHV infection. HUVECs were first transduced with lentiviruses of properdin-specific shRNAs and selected with puromycin for 10 days. The cells were then infected with KSHV for 24 h and the C5b-9 deposition was analyzed. Properdin-specific shRNAs reduced the expression of properdin protein (Figure 7A) and its secretion to the extracellular media (Figure 7B). In agreement with these results, C5b-9 deposition was significantly suppressed in acute KSHV-infected cells following the knock down of properdin (Figure 7C). Together, these results indicated that properdin was a crucial factor for complement activation during *de novo* KSHV infection.

### Terminal complement complexes suppress KSHV lytic replication during acute infection of endothelial cells

**Figure 9:**
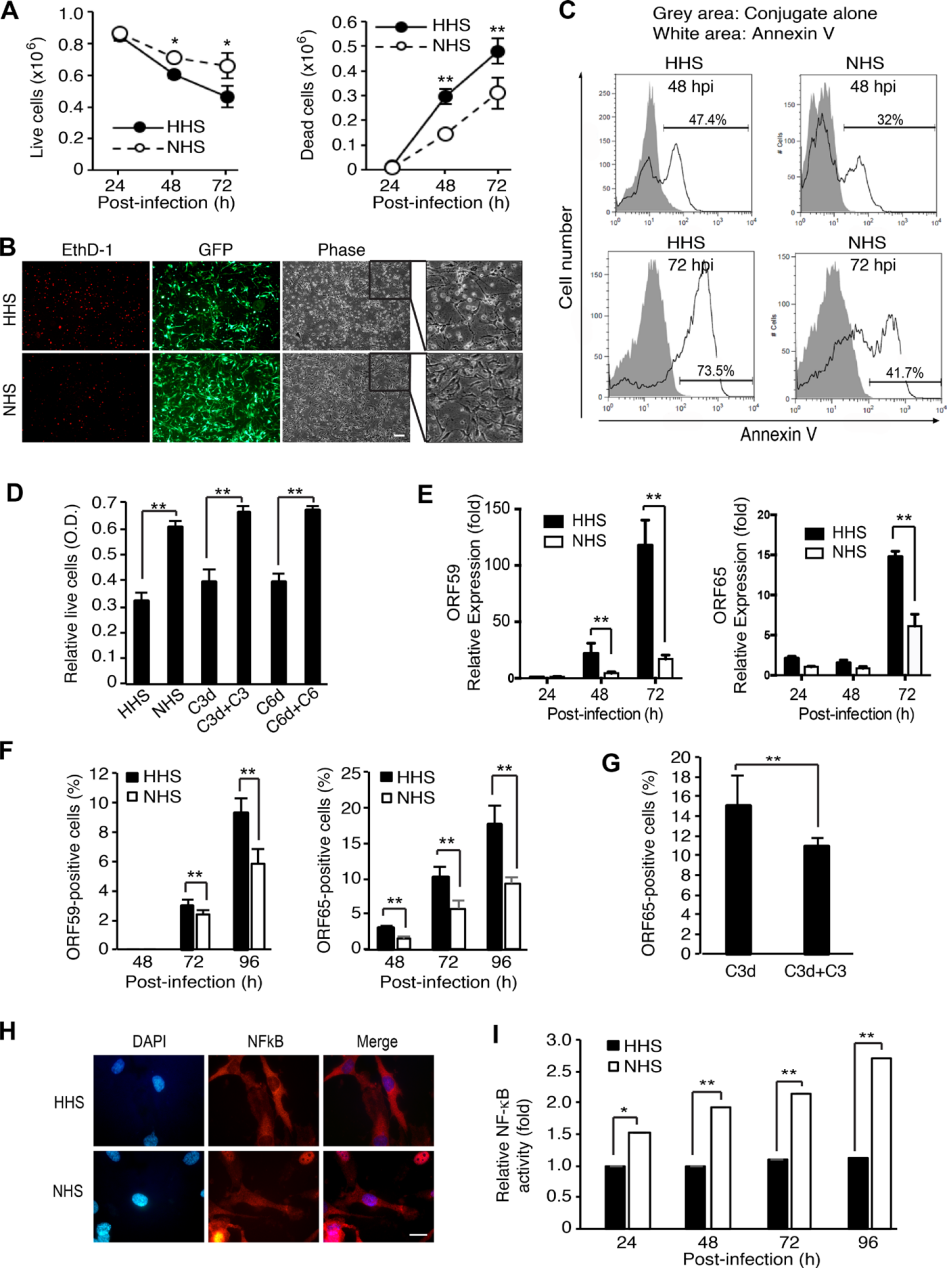
Complement activation confers a survival advantage to KSHV-infected endothelial cells during acute KSHV infection (**A**–**C**) Complement activation promotes cell survival during acute KSHV infection of human endothelial cells. HUVECs infected with KSHV in the presence of heat-inactivated serum (HHS) or normal human serum (NHS) were examined for live and dead cells by trypan blue exclusion assay at the indicated time points (A), stained with the cell-impermeant viability indicator ethidium homodimer-1 (EthD-1) at 48 hpi (B) or examined for apoptosis by flow cytometry following Annexin V staining at the indicated time points (C). Scale bar: 100 μm. (**D**) Exogenous complement factors C3 and C6 are required for complement promotion of cell survival during KSHV acute infection. HUVECs infected with KSHV in the presence of HHS, NHS, C3-depleted human serum (C3d), C3-depleted human serum compensated with recombinant C3 (C3d + C3), C6-depleted human serum (C6d), or C6-depleted human serum compensated with recombinant C6 (C6d + C6) were examined for relative cell viability at 72 hpi by WST-1 assay. (**E**–**F**) Expression of KSHV lytic genes was suppressed by complement activation. HUVECs infected with KSHV in the presence of HHS or NHS were examined for the expression of ORF59 and ORF65 at the indicated time points by RT-qPCR (E) and immunofluorescence assay (IFA, F). Percentages of positive cells in IFA were calculated based on 10 microscopic fields of view (40x) from three independent experiments. (**G**) Complement factor C3 is required for inhibition of KSHV lytic replication during acute infection of HUVECs. HUVECs infected with KSHV in the presence of C3-depleted human serum (C3d) or C3-depleted human serum compensated with recombinant C3 (C3d + C3) were examined for ORF65-poasitive cells at 72 hpi by IFA. (**H**–**I**) Complement activation activates the NF-κB pathway during acute KSHV infection of human endothelial cells. HUVECs infected with KSHV in the presence of HHS or NHS were examined for the translocation of NF-κB in IFA (H) and relative NF-κB activity at the indicated time points by measuring the DNA-binding activity of NF-κB using an Ez-Detect transcription factor kit for NF-κB p65 (I). Scale bar: 20 μm. Results are shown as means ± standard deviations, ^*^p < 0.05 and ^**^p < 0.01.

Complement activation can lead to cell lysis, which is effectuated by MAC. However, sublytic complement activation is frequently associated with enhanced cell survival [38, 39]. We investigated the effect of complement activation on cell survival during KSHV acute infection. Compared to HHS-treated cells, NHS-treated cells showed significantly higher numbers of live cells and lower numbers of dead cells at 48 and 72 hpi (Figure 9A). We observed a greater number of EthD-1-positive dead cells and floating cells in HHS- than NHS-treated cultures (Figure 9B). Because dead cells lose their GFP signals, fewer GFP expressing cells were observed among HHS-treated cells than NHS-treated cells. There were also more Annexin V-positive apoptotic cells in HHS- than NHS-treated cultures (Figure 9C). Together, these results indicated that complement activation conferred a survival benefit to KSHV-infected endothelial cells during acute infection.

The complement system regulates cell survival through several mechanisms. Activation of the complement system promotes survival signals as a result of the interaction of C3a and C5a with their receptors [39] as well as binding of terminal complement complexes to cell surfaces. To determine which phase of complement activation promoted cell survival during KSHV acute infection, we analyzed cell survival in KSHV infected cells treated with human serum depleted of complement factor C3 or C6 (Figure 9D). Depletion of C3 or C6 completely abolished the enhancement of cell survival by NHS while addition of C3 or C6 to the respective depleted human serum rescued cell survival (Figure 9D). These results indicated that complement promotion of cell survival during KSHV acute infection was mediated by terminal complement complexes in the late phase of the complement cascade.

Previous studies have shown that KSHV acute infection of HUVECs is productive resulting in substantial cell death [14, 15]. Therefore, we investigated whether the observed enhanced cell survival by complement was due to inhibition of viral lytic replication. Indeed, mRNA expression and the numbers of ORF65- and ORF59-positive cells was significantly reduced in KSHV-infected HUVECs treated with NHS compared to those treated with HHS (Figure 9E, 9F and Supplementary Figure 6). Depletion of C3 from human serum significantly increased the number of ORF65-positive cells while addition of C3 to the C3-depleted human serum reversed the effect (Figure 9G), confirming that the reduced viral lytic replication was mediated by the activated complement pathway.

C5b-9 is known to activate the transcription factor NF-kB in various cells, including human endothelial cells [40, 41]. Several studies have reported that activation of NF-kB pathway inhibits KSHV lytic replication [42, 43]. Thus, we hypothesized that complement activation might activate the NF-kB pathway resulting in the inhibition of KSHV lytic replication. Indeed, we detected the nuclear translocation of NF-kB (Figure 9H) and higher NF-kB activities in KSHV-infected HUVECs treated with NHS than those treated with HHS in the DNA-binding activity of NF-kB assay (Figure 9I), which is consistent with results of a previous study [41].

Taken together, these results indicated that complement activation promoted cell survival by activating the NF-kB pathway to suppress viral lytic replication during KSHV acute infection of HUVECs.

## DISCUSSION

In this study, we have described a novel role of EVs from *de novo* KSHV-infected human endothelial cells in complement activation. The complement activation by KSHV-induced EVs is mediated by the alternative complement pathway, which is associated with the activation of endogenous C3 and properdin.

Viruses and EVs can have a biological effect on the recipient cells and the microenvironment [3, 44]. Increased evidences indicated that viruses and EVs share the similar host biogenesis pathways [45, 46]. Therefore, understanding the biogenesis of EVs and their functions in viral infection is important for gaining new insights into viral pathogenesis, and this has been the topic for a number of recent reviews [47, 48]. Several studies have reported that herpesviruses modulate host EVs for their own benefit [13, 49]. KSHV has also been shown to alter the proteins and microRNAs in the exosomes secreted from latent KSHV-infected B cells [11, 12]. However, the role of EVs in viral infection and the mechanism of action are largely unknown.

The complement system is an innate immune effector that protects against common pathogens. Physiologically, complements can induce an inflammatory reaction, opsonization, and membrane attack complex (MAC)-mediated cell lysis [21]. In addition to the roles in innate immune defense against pathogens, complements participate in diverse immunological and inflammatory processes, including control of adaptive immunity, removal of apoptotic cells, and regulation of the coagulation system. Furthermore, recent studies indicate that the complement system has various effects on cell survival and tumor progression [50, 51]. Since KSHV encodes a complement inhibitor KCP, the complement system is believed to be suppressed during KSHV infection [52]. We have shown that during latent KSHV infection, most viral lytic proteins including KCP are not expressed but the complement system is activated as a result of the suppression of cellular complement regulatory proteins [32, 53]. However, there have been no reports on the status of the complement system during *de novo* KSHV infection so far. In this study, we have surprisingly found that the complement system is activated by EVs during *de novo* KSHV infection of human endothelial cells despite the upregulation of complement regulatory proteins.

Properdin is synthesized by numerous cells such as monocytes, dendritic cells, primary T cells, mast cells, and adipocytes [54]. Multiple inflammatory agonists stimulate the release of properdin into pro-inflammatory microenvironments to locally activate the complement system [54]. Endothelial cells are not a major source of properdin but a previous study has shown that HUVECs constitutively express a small amount of properdin and that laminar shear stress could further stimulate the production of properdin [35]. Intriguingly, our study has shown that KSHV infection induces cell surface expression of properdin in the infected endothelial cells. Most importantly, we have shown that properdin is essential for complement activation during *de novo* KSHV infection, suggesting the presence of a novel pathway of complement activation by cell surface properdin. A previous study has shown that endogenous native properdin in neutrophils can be detected on cell surfaces and can trigger complement activation [55]. These observations are in agreement with our results that complement activation is mediated by direct binding of properdin to cell surfaces.

Our study demonstrated that KSHV infection induces complement activation not only in the infected cells but also in the neighboring cells. This autocrine and paracrine effect is mediated by EVs released from KSHV-infected HUVECs albeit the exact factor responsible for this remains to be elucidated. EVs mediate intercellular communication by serving as vehicles to transfer membrane and cytosolic proteins, lipids, and RNAs, including mRNAs, microRNAs, and various small noncoding RNAs between cells [2]. A previous study suggested that exosomes isolated from latent KSHV-infected cells might participate in paracrine signaling [11, 12]. Here we showed a function for EVs released from KSHV-infected primary endothelial cells, which is to induce the activation of the complement system. As EVs-mediated complement activation is triggered during the entry and trafficking of KSHV, this may be considered as an innate immune response in the cells against virus infection. It would be interesting to investigate whether complement activation in infections of other viruses is mediated EVs.

Despite the complement system is activated during acute KSHV infection, it does not prevent KSHV infection. The complement system is generally recognized as a primary defense mechanism against pathogens [56]. During a viral infection, the activated complement system inhibit the viral infection or eradicate virus-infected cells [57]. However, we have not observed any adverse effects of complement activation on KSHV infection; rather, the activated complement promotes cell survival as a result of activating the NF-kB pathway, and KSHV switches to latency by turning down the lytic replication in response to the activated NF-kB pathway, both of which enhance persistent viral infection. Because complement factors interact with various other immune components, further studies might reveal additional mechanisms of KSHV activation of the complement system, and its role in KSHV infection.

KS is a highly inflammatory tumor. The extent to which complement activation during KSHV *de novo* infection contributes to the tumor inflammatory microenvironment remains to be elucidated. Spontaneous viral lytic replication in a small subset of KSHV-infected tumor cells is often observed in the early stage of KS tumors, which could lead to the production of infectious virions [9]. The resulting *de novo* infection not only directly induces inflammatory cytokines [58, 59] but also activates the complement system through EVs. Thus, KSHV *de novo* infection and the resulting complement activation could contribute to the inflammatory KS tumor microenvironment and KS pathogenesis. Collectively, our findings provide novel and important insights on the role of EVs and their mechanisms of action during KSHV infection.

## MATERIALS AND METHODS

### Vectors, cell cultures, and reagents

HUVECs were purchased from Lonza (Allendale, NJ), and cultured with endothelial cell growth medium-2 (EGM-2) bullet kit (Lonza) in a humidified atmosphere of 5% CO_2_ at 37°C. Pooled complement human serum was purchased from Innovative Research, Inc (Novi, MI) and used as normal human serum (NHS) in all experiments. Heat-inactivation was performed with this serum at 56°C for 30 min. Factor B-depleted human serum, C3-depleted human serum, C6-depleted human serum, purified properdin, purified C3 and purified factor B were purchased from Quidel Corporation (San Diego, CA). Factor P (properdin)-depleted human serum was purchased from Complement Technology, Inc. (Tyler, Tx). EDTA, EGTA, heparin, GW4869, methyl-β–cyclodextrin, chlorpromazine, and phosphonoformic acid were obtained from Sigma-Aldrich (St. Louis, MO).

### Virus isolation and infection

iSLK BAC16 cells harboring recombinant KSHV BAC16 were used as the source of virus [19]. Infectious KSHV BAC16 virions were induced from iSLK BAC16 cells by treatment with doxycycline and sodium butyrate for three days. The culture supernatant was collected, filtered through a 0.22 μm filter, and centrifuged at 100,000 ×*g* for 1 h. The pellet was resuspended in phosphate-buffered saline (PBS) and stored at −70°C as infectious virus particles. HUVECs were infected with KSHV according to methods used in a previous study [60]. iSLK BAC16 and extracted virus are periodically checked for mycoplasma infection by PCR using specific primers.

### Immunofluorescence assay

Cells were seeded onto a microscopy cover glass in 24-well tissue culture plates at a density of 1 × 10^5^ cells/well. After culturing overnight, culture media was removed and washed with PBS. The cells were fixed with 4% paraformaldehyde and blocked with 3% bovine serum albumin (BSA) in PBS. Rabbit polyclonal anti-C5b-9 (Abcam, Cambridge, MA), mouse monoclonal anti-C3b (Thermo Scientific, Rockford, IL), or rabbit polyclonal anti-properdin (Bioss Antibodies Inc., Woburn, MA) were used as primary antibodies. Cells were incubated with a primary antibody, then incubated with Alexa Fluor-conjugated goat anti-rabbit or goat anti-mouse antibody (Invitrogen, Carlsbad, CA). Nuclei were stained using 4,6-diamidino-2-phenylindole (DAPI). Cells were mounted with Vectashield^®^ (Vector Laboratories Inc., Burlingame, CA) and examined using an Eclipse E400 microscope (Nikon Instruments Inc., Melville, NY). Images were captured using a Nikon Digital site Fi3, and analyzed using NIS element BR.

### Lentivirus infections

Plasmids containing shRNAs for human properdin (TRCN0000377701 and TRCN0000084069, Sigma, St. Louis, MO) or a scramble shRNA (#1864, Addgene, Cambridge, MA) were co-transfected with pPACKF1 packaging plasmid mix (SBI, Palo Alto, CA) into HEK293T cells using Lipofectamine 3000 transfection reagent (Thermo Scientific) as per the manufacturer’s recommendations. HUVECs were infected with viral supernatants from HEK293T cells along with polybrene (5 μg/mL) for 24 h. After 10 days of selection with puromycin (0.5 μg/mL), efficiency of properdin knockdown was evaluated by ELISA and western blotting.

### Detection of C5b-9 by cell-based ELISA

Quantification of C5b-9 with a cell-ELISA technique was performed as previously described [20].

### Flow cytometry

To detect cell surface C3b, HUVECs were treated with culture media containing 10% pooled human serum before trypsinization. The cells were detached from the plate by trypsinization and incubated with primary antibodies for 30 min on ice before being washed three times with blocking solution (1% FBS in PBS) and labeled with allophycocyanin (APC)-conjugated secondary antibody (BD Biosciences, San Jose, CA) for 30 min at 4°C. Mouse monoclonal anti-C3b antibody (Thermo Scientific), rabbit polyclonal CD46 antibody (Santa Cruz Biotechnology, Dallas, Tx), rabbit polyclonal anti-CD55 (Santa Cruz Biotechnology), or rabbit polyclonal anti-CD59 antibody (Abcam) was used as primary antibody. After three additional washes, the cells suspended in blocking solution were analyzed with a Guava Easycyte Flow Cytometer and InCyte 3.1 software (Merck Millipore, Bedford, MA). For analysis of apoptosis, Annexin V apoptosis detection kit APC (eBioscience, San Diego, CA) was used according to the manufacturer’s recommendations.

### Detection of virion DNA

The supernatants of KSHV-infected HUVECs were collected and centrifuged at 100,000 × *g* for 1 h. The pellet was resuspended in 1X DNase buffer and then treated by RQ1 RNase-free DNase I (Promega, Madison, WI) at 37°C for 1 h. DNA was extracted using the QIAamp DNA blood minikit (Qiagen, Hilden, Germany) according to the manufacturer’s recommendations, and PCR analysis was carried out using the PCR premix (Enzynomics, Daejeon, South Korea) with primers ORF26F (5’ GAC TCT TCG CTG ATG AAC TGG 3’) and ORF26R (5’ AGC ACT CGC AGG GCA GTA CG 3’) targeting KSHV ORF26 (Genotech, Daejun, South Korea). Reactions were performed for 35 cycles of 30 s at 95°C, 30 s at 50°C, and 60 s at 72°C. The amplified products were analyzed on a 2% agarose gel by electrophoresis.

### Western blotting

Western blotting was performed as described previously with modifications [32]. Cells or EVs were lysed in 1x RIPA buffer with protease inhibitors. The lysate was centrifuged and the supernatants collected. Mouse monoclonal anti-beta-actin (Sigma), mouse monoclonal anti-C3b (Thermo scientific), mouse monoclonal anti-properdin (Abcam), rabbit monoclonal anti-HSP70 (Abcam), rabbit polyclonal anti-CD59 (Abcam), rabbit polyclonal anti-CD55 (Santa Cruz Biotechnology), mouse monoclonal anti-CD63 (Santa Cruz Biotechnology), mouse monoclonal anti-CD81 (Santa Cruz Biotechnology), Rabbit polyclonal anti-CD46 (Santa Cruz Biotechnology), Rabbit polyclonal anti-Calnexin (Bioss Antibodies Inc.), and rabbit polyclonal anti-CD55 (Santa Cruz Biotechnology) were used.

### ELISA for properdin and exosome markers

Secreted properdin was measured using a human properdin ELISA kit according to the manufacturer’s instructions (Hycult Biotech, Plymouth Meeting, PA). To measure levels of exosome marker HSP70, samples containing EVs were diluted in 0.1 M sodium bicarbonate buffer (pH 9.6) and used to coat each well in a 96-well EIA plate. After washing with Tris-buffered saline (TBS) containing 0.05% Tween-20 (TBS-T), the plates were incubated with mouse monoclonal anti-Hsp70 (Abcam). Plates were washed three times with TBS-T for 15 min, and incubated with HRP-conjugated anti-mouse IgG (Santa Cruz Biotechnology). Following washing, the plates were developed using as a substrate and the absorbance was measured at 450 nm using a microplate reader (Molecular Devices, Sunnyvale, CA).

### Extraction and purification of EVs

EVs were isolated from the supernatant of mock- or KSHV-infected HUVECs at 24 hpi by differential ultracentrifugation. Briefly, cells were removed from conditioned medium by centrifugation at 300 × *g* for 10 min and the collected medium was filtered through 0.22-μm filter, followed by ultracentrifugation at 100,000 × *g* for 60 min at 4°C. The supernatant was removed and the pellet was resuspended in PBS for overnight at 4°C. Isolated EVs were stored at 4°C and used for experiments within 2 weeks. Isolation of EVs by ExoQuick-TC (SBI) was performed as manufacturer’s recommendation. Briefly, 10 mL of conditioned medium from the cell culture was mixed with ExoQuick-TC solution and incubated overnight at 4°C. After centrifugation (1,500 × *g* for 30 min), supernatants were discarded, and tubes were centrifuged again (1,500 × *g* for 5 min). All traces of fluid were aspirated, and the pellets were resuspended in 200 μL of PBS. For ultrafiltration methods, conditioned media were fractionated using Vivaspin 20 protein concentrator spin columns with molecular weight cut-offs of 100, 50, and 10 kDa (GE Healthcare Life Sciences, Pittsburgh, PA) as recommended by the manufacturer.

### Nanoparticle tracking analysis

The numbers of microparticles in EVs preparations were analyzed by nanoparticle tracking analyzer, ZetaView (Particle Metrix GmbH, Meerbusch, Germany). Preparations of EVs were diluted in PBS and passed through 0.22 µm filters before the analysis. The analysis parameters were as follows: max size 200, min size 20, brightness 20, sensitivity 75, and temperature 25°C.

### Protein digestion and nano-ESI-LC-MS/MS analysis

Samples of EVs were re-constituted in buffer containing 8 M urea and 0.1 M Tris-HCl. Protein concentration of the EVs lysate was measured using a Bradford assay (Bio-Rad, Hercules, CA). Protein samples (250 μg) were reduced by treatment with 5 mM Tris(2-carboxyethyl)phosphine (Pierce, Rockford, IL, USA) at 37°C for 30 min and alkylated with 15 mM iodoacetamide at room temperature for 60 min. Then, the samples were digested with mass-spectrometry grade trypsin gold (Promega, Rockford, IL, USA) at 37°C overnight. Peptides were desalted on a Sep-Pak C18 cartridge (Waters, Milford, MA, USA) and separated into 5 fractions based on their isoelectric point via OFFGEL fractionator (Agilent Technologies, Santa Clara, CA, USA). Each peptide fraction was analyzed using a high-performance liquid chromatography (HPLC)-chip/quadrupole time-of-flight system (Agilent Technologies). The chromatography parameters were as follows: 160 nL enrichment column; 75 μm × 150 mm separation column packed with Zorbax 300SB-C18 (5 μm); flow rate, 0.4 μL/min. Eluted peptides were selected for collision-induced dissociation during alternative events of an MS scan over the m/z range of 300–2400 at the rate of 4 spectra/s, and an MS/MS scan over the range of 100–3000 m/z at 3 spectra/s.

### Protein identification and bioinformatics analysis

All tandem mass spectra were searched by Spectrum Mill against the SwissProt database. Parameters were set with a precursor mass tolerance of 20 ppm, maximum missed cleavage site of 2, fixed modification of carbamidomethyl cysteine and variable modifications of oxidized methionine. The identified proteins were investigated for similarities and differences in features for label-free quantification using Mass Profiler Professional (MPP) software (Agilent Technologies). After normalization, target features showing more than 2-fold intensity differences were selected and denoted as differentially expressed proteins (Supplementary Table 1). The proteins were annotated and classified based on their molecular functions using PANTHER (Protein Analysis Through Evolutionary Relationships) software. The classifications of uploaded proteins were reviewed, and minor changes were obtained based on published data regarding molecular function (NCBI OMIM; http://www.ncbi.nlm.nih.gov/entrez/query.fcgi?db=OMIM). Proteins that did not matches to entry in the PANTHER database or have no annotated molecular function were classified to one of PANTHER’s parent groups by considering published data on protein molecular function (NCBI OMIM). GeneGo MetaCore software (ISB, Seattle, WA, USA) was used to analyze pathway map of differentially expressed proteins. The ranked pathway maps were created based on the calculated *p*-values.

### Electron microscopy

Samples were analyzed by transmission electron microscopy using negative staining. Five microliters of sample was loaded on glow discharge carbon coated copper mesh grids, and stained with 2% uranyl acetate in aqueous solution. Samples were examined with a Hitachi 7600 transmission electron microscope.

### NF-kB p65 transcription factor assay

The DNA-binding activity of NF-kB was assessed using an EZ-Detect transcription factor kit for NF-kB p65 following the manufacturer’s instructions (Thermo Scientific). Samples were prepared with RIPA lysis buffer. DNA binding specificity was assessed using wild-type and mutant NF-kB oligonucleotides. Chemiluminescent detection was performed using a luminometer (PerkinElmer, Hopkinton, MA).

### Statistical analysis

Results are shown as means standard deviations. The one-tailed Student’s *t* test was used to assess the significance of the difference between groups. Statistical significance at *P* values of < 0.05 and < 0.01 is indicated by ^*^ and ^**^, respectively.

## SUPPLEMENTARY MATERIALS FIGURES AND TABLE




