# Supplementary material for: Extracellular vesicles from KSHV-infected endothelial cells activate the complement system
Source: Oncotarget. 2017 Oct 9;8(59):99841–60. doi: 10.18632/oncotarget.21668 (PMC5725135; doi:10.18632/oncotarget.21668)
Supplement: Supplementary file 1 [file oncotarget-08-99841-s001.pdf]

Extracellular vesicles from KSHV-infected endothelial cells activate the complement system

SUPPLEMENTARY MATERIALS

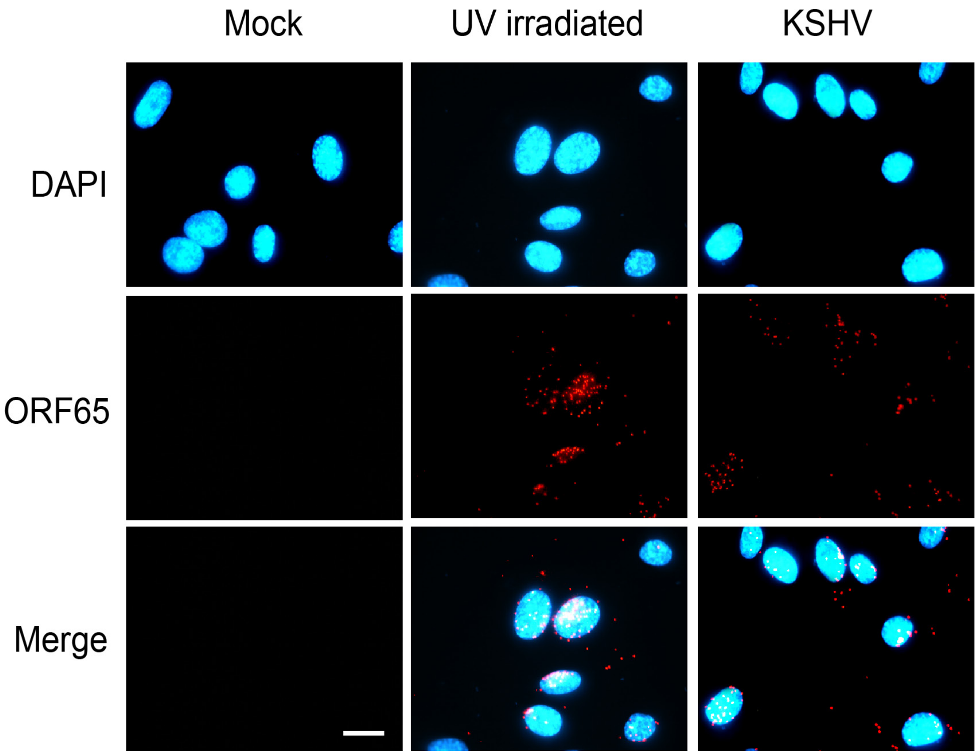

Supplementary Figure 1: UV irradiation does not affect KSHV entry and trafficking into HUVECs. HUVECs were infected with KSHV or UV-irradiated KSHV for 4 hours and stained for KSHV particles by IFA using a monoclonal antibody to ORF65. Nuclei were stained with DAPI. Scale bar: 20 µm.

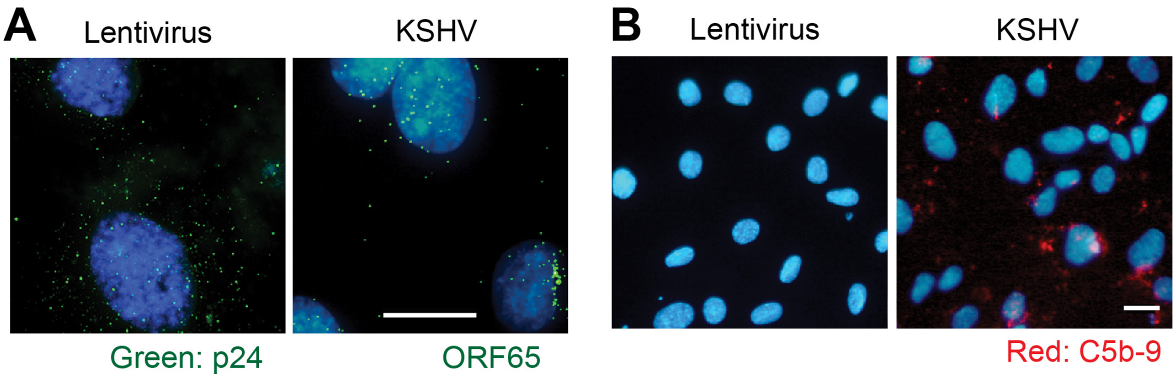

Supplementary Figure 2: Not all virus-infected cells activate the complement system. (**A**) Entry of lentivirus into human endothelial cells. HUVECs were infected with lentivirus or KSHV. Viral particles were visualized by IFA. Lentivirus and KSHV particles was detected at 4 hpi with anti-HIV p24 and anti-KSHV ORF65 antibody, respectively (green). Nuclei were stained with DAPI (blue). Scale bar: 20 µm. (**B**) C5b-9 deposition was not detected in lentivirus-infected HUVECs. At 24 hpi, lentivirus-infected or KSHV-infected HUVECs were treated with NHS for 30 min. The deposition of C5b-9 was analyzed by IFA. Scale bar: 20 µm.

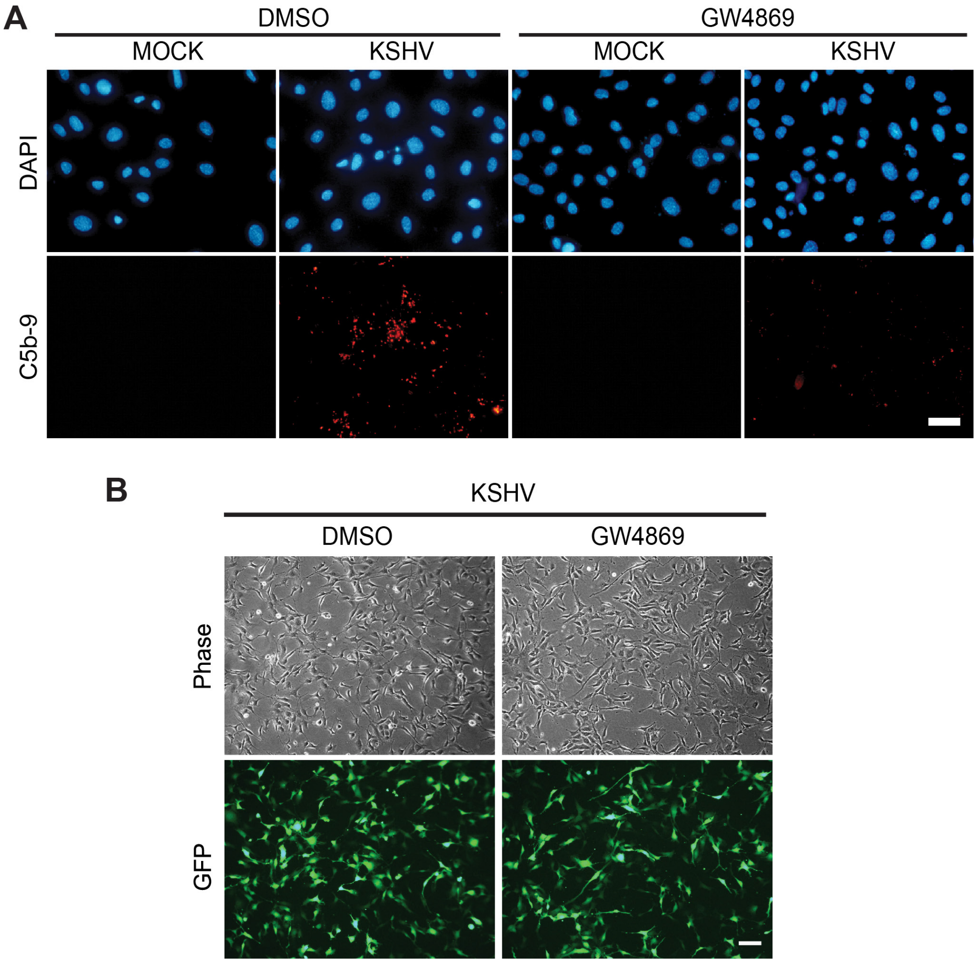

Supplementary Figure 3: Inhibition of EVs biogenesis during KSHV infection impairs complement activation by EVs from KSHV-infected cells. (**A**) HUVECs pretreated with dimethyl sulfoxide (DMSO) or 10 µM GW4869 for 1 h were either mock-infected or infected with KSHV in the presence of respective agents. The conditioned media (CM) collected at 24 hpi was added to naïve HUVECs and C5b-9 deposition was examined by IFA. Before analysis of C5b-9 deposition, HUVECs were exposed to normal human serum for 30 min. Mock: conditioned media collected from mock-infected HUVECs; KSHV: conditioned media collected from KSHV-infected HUVECs. Scale bar: 50 µm. (**B**) Green fluorescent protein (GFP) expression of KSHV-infected HUVECs pretreated with DMSO or GW4869 before EVs isolation. Scale bar: 100 µm.

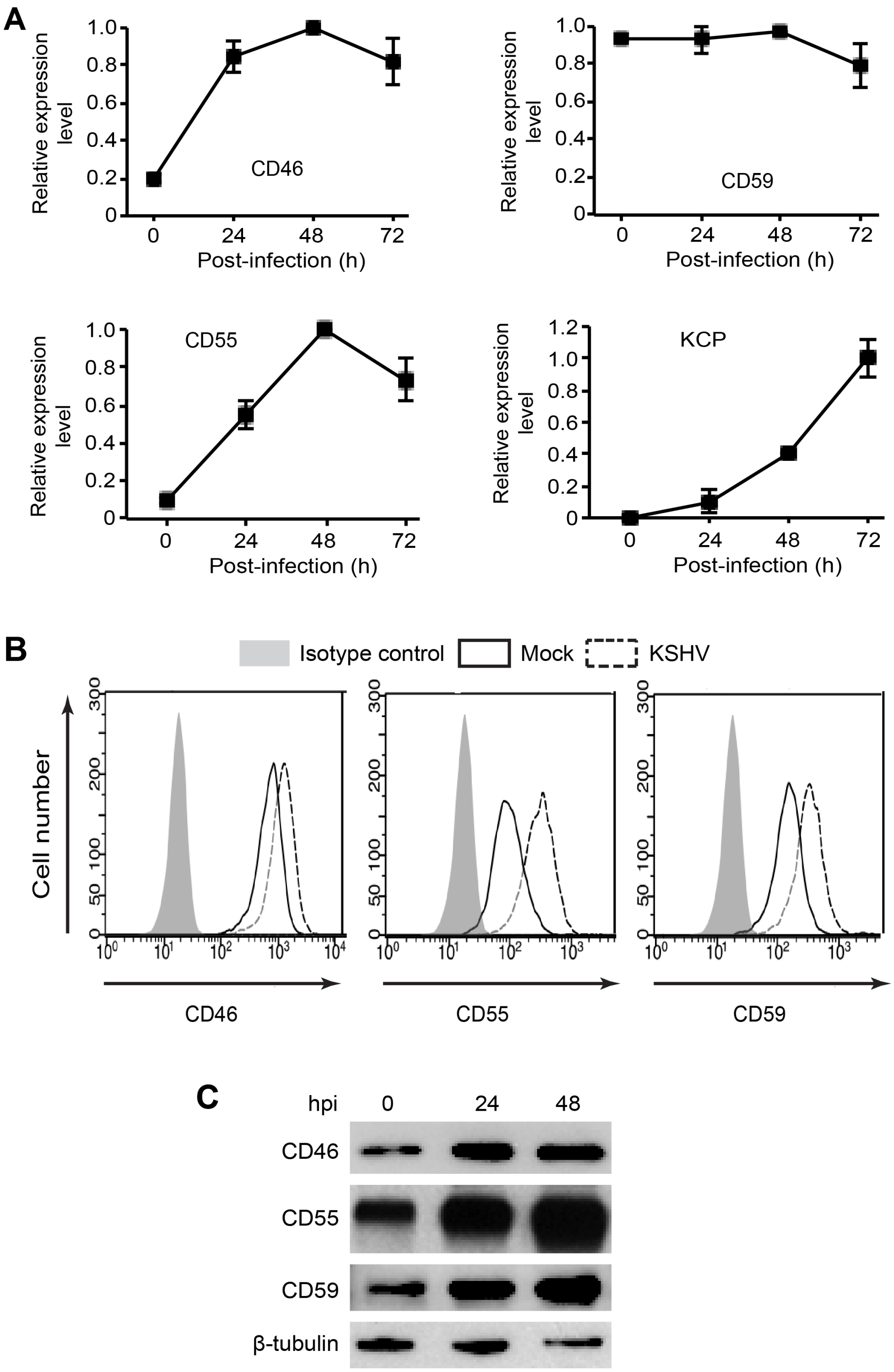

Supplementary Figure 4: Expression of complement regulatory proteins and KSHV complement control protein (KCP) during de novo KSHV infection of HUVECs. (**A**) Time kinetics for mRNA expression of complement regulatory proteins and KCP in HUVECs during acute KSHV infection examined by RT-qPCR. (**B**–**C**) Expression of CD46, CD55, and CD59 proteins in HUVECs at the indicated time points after KSHV infection measured by flow cytometry (B) and western blotting (C).

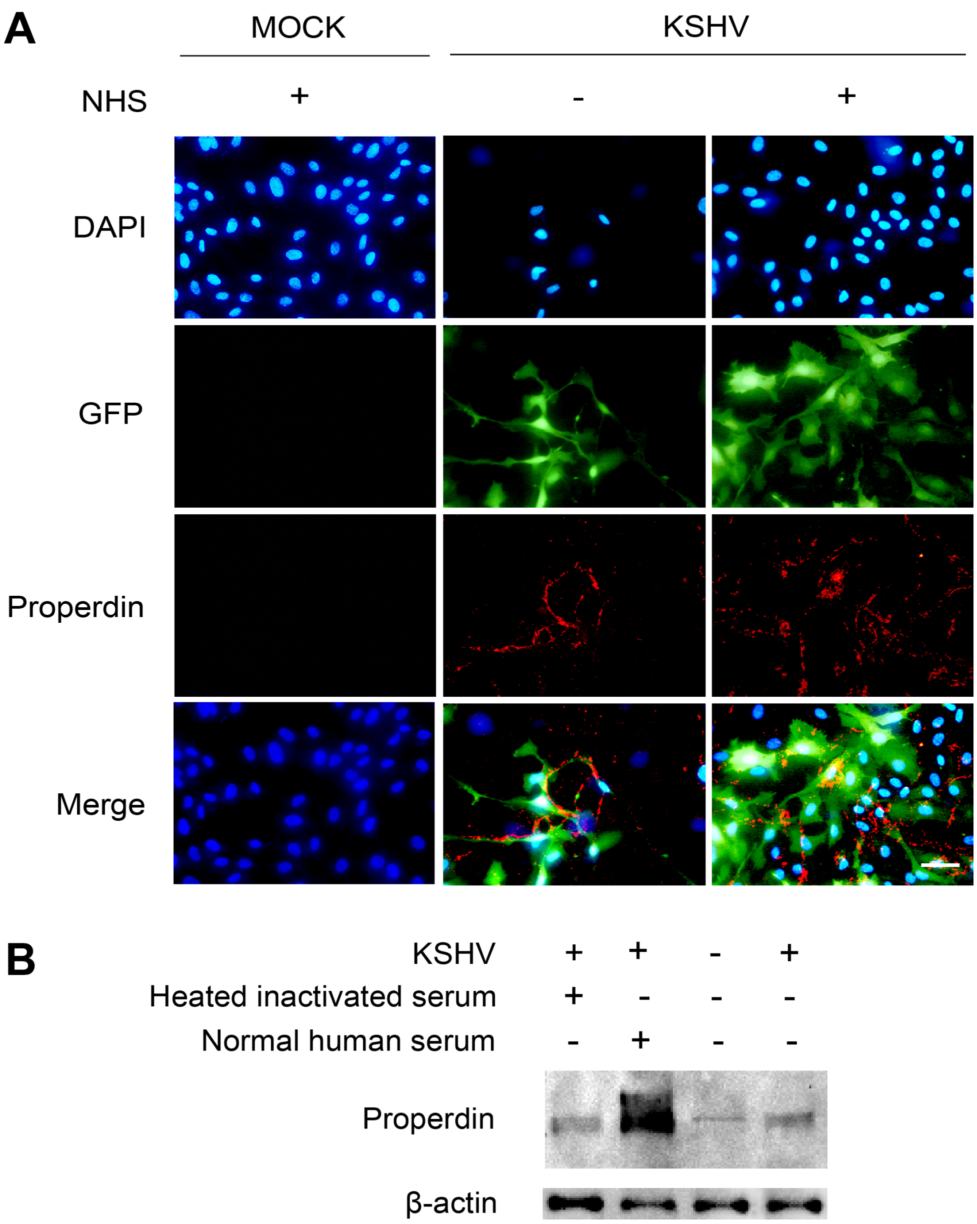

Supplementary Figure 5: Detection of properdin in KSHV-infected HUVECs. Properdin was detected in de novo KSHV-infected HUVECs at 24 hpi by IFA (**A**) and western blotting (**B**). Scale bar: 50 µm.

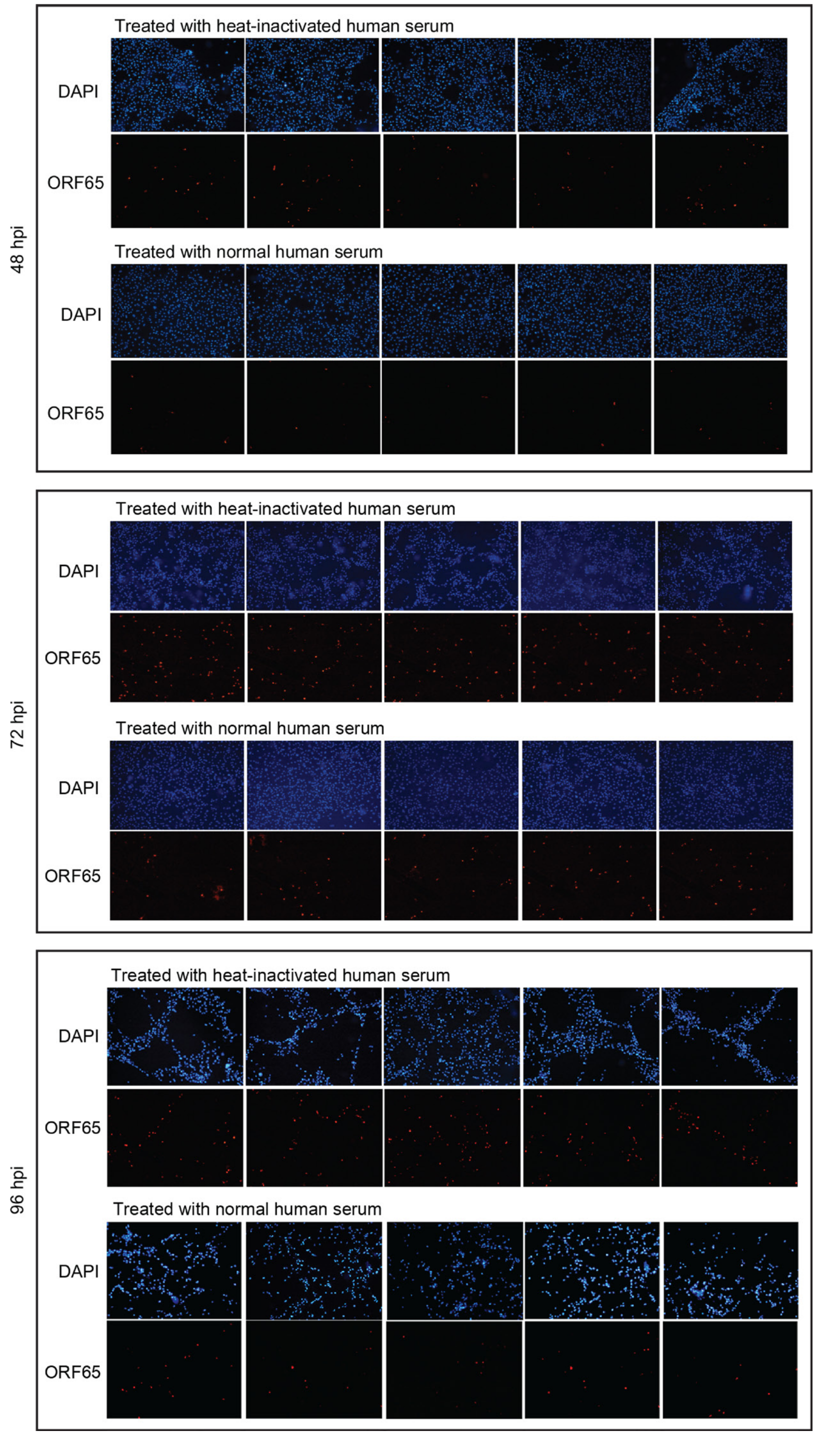

Supplementary Figure 6: Expression of KSHV lytic genes was suppressed by complement activation. HUVECs infected with KSHV in the presence of HHS or NHS were examined for ORF65-positive cells at the indicated time points by immunofluorescence assay (IFA). Magnification, 40x.

Supplementary Table 1: Differentially expressed proteins in virus compared with non-virus. See Supplementary_Table_1
