# Supplementary material for: Extracellular vesicles from KSHV-infected endothelial cells activate the complement system
Source: Oncotarget. 2017 Oct 9;8(59):99841–60. doi: 10.18632/oncotarget.21668 (PMC5725135; doi:10.18632/oncotarget.21668)
Supplement: Supplementary file 2 [file oncotarget-08-99841-s002.docx]

**Supplementary Table 1: Differentially expressed proteins in virus compared with non-virus**

| **Protein name** | **Accession number*** | **Fold change** | **Change of expression level^†^** |
| --- | --- | --- | --- |
| Gelsolin | P06396 | 171 | Increased |
| Tubulin beta-4B chain | P68371 | 1295 | Increased |
| Tubulin beta chain | P07437 | 277 | Increased |
| Serglycin | P10124 | 1650 | Increased |
| 60S ribosomal protein L13 | P26373 | 3 | Increased |
| Fibulin-1 | P23142 | 2 | Increased |
| Hemoglobin subunit epsilon | P02100 | 2 | Increased |
| 60S acidic ribosomal protein P1 | P05386 | 2 | Increased |
| Thrombospondin-4 | P35443 | 2 | Increased |
| Lipoxygenase homology domain-containing protein 1 | Q8IVV2 | 238 | Increased |
| Fibromodulin | Q06828 | 9 | Increased |
| Kinesin-like protein KIF7 | Q2M1P5 | 3 | Increased |
| Transforming growth factor-beta-induced protein ig-h3 | Q15582 | 198 | Increased |
| Melanoma-associated antigen 4 | P43358 | 11 | Increased |
| Protein Shroom3 | Q8TF72 | 82 | Increased |
| Protein disulfide-isomerase A3 | P30101 | 9 | Increased |
| Myosin-10 | P35580 | 264 | Increased |
| Phosphatidylinositol 3,4,5-trisphosphate-dependent Rac exchanger 2 protein | Q70Z35 | 1132 | Increased |
| Kelch-like protein 32 | Q96NJ5 | 81 | Increased |
| WD repeat-containing protein 72 | Q3MJ13 | 2 | Increased |
| Putative uncharacterized zinc finger protein 814 | B7Z6K7 | 2 | Increased |
| NADH dehydrogenase [ubiquinone] iron-sulfur protein 3, mitochondrial | O75489 | 3 | Increased |
| Uncharacterized protein KIAA2012 | Q0VF49 | 2 | Increased |
| Probable E3 ubiquitin-protein ligase HERC1 | Q15751 | 2 | Increased |
| Alpha-enolase | P06733 | 150 | Increased |
| Inter-alpha-trypsin inhibitor heavy chain H1 | P19827 | 147 | Increased |
| WD repeat-containing protein 87 | Q6ZQQ6 | 6 | Increased |
| Nuclear factor of activated T-cells 5 | O94916 | 3 | Increased |
| Gap junction delta-4 protein | Q96KN9 | 6 | Increased |
| Heparin cofactor 2 | P05546 | 3 | Increased |
| Zinc finger protein 518B | Q9C0D4 | 329 | Increased |
| Complement component C9 | P02748 | 9 | Increased |
| ICOS ligand | O75144 | 6 | Increased |
| Nidogen-1 | P14543 | 168475 | Increased |
| Heat shock protein beta-1 | P04792 | 54 | Increased |
| Putative ATP-dependent RNA helicase TDRD9 | Q8NDG6 | 8249 | Increased |
| E3 SUMO-protein ligase RanBP2 | P49792 | 4931 | Increased |
| Voltage-dependent T-type calcium channel subunit alpha-1I | Q9P0X4 | 8871 | Increased |
| Ankyrin repeat domain-containing protein 53 | Q8N9V6 | 161 | Increased |
| Eukaryotic initiation factor 4A-II | Q14240 | 64 | Increased |
| Adenosine deaminase-like protein | Q6DHV7 | 164 | Increased |
| Syntenin-1 | O00560 | 126 | Increased |
| Talin-1 | Q9Y490 | 61 | Increased |
| Ras GTPase-activating-like protein IQGAP2 | Q13576 | 164563 | Increased |
| Mitogen-activated protein kinase kinase kinase 4 | Q9Y6R4 | 237 | Increased |
| Septin-9 | Q9UHD8 | 1089 | Increased |
| Myotubularin-related protein 1 | Q13613 | 30 | Increased |
| Apolipoprotein E | P02649 | 31 | Increased |
| Putative uncharacterized protein MYH16 | Q9H6N6 | 181 | Increased |
| Leucine zipper putative tumor suppressor 3 | O60299 | 211 | Increased |
| E3 ubiquitin-protein ligase HERC2 | O95714 | 183 | Increased |
| Nuclear distribution protein nudE homolog 1 | Q9NXR1 | 273 | Increased |
| Neuron navigator 3 | Q8IVL0 | 451 | Increased |
| Ephrin type-A receptor 2 | P29317 | 80084 | Increased |
| AT-hook DNA-binding motif-containing protein 1 | Q5TGY3 | 516 | Increased |
| Leucine-rich repeat and fibronectin type III domain-containing protein 1 | Q9P244 | 207 | Increased |
| Phosphoribosylformylglycinamidine synthase | O15067 | 31 | Increased |
| TBC1 domain family member 1 | Q86TI0 | 199 | Increased |
| Serine/threonine-protein kinase mTOR | P42345 | 260 | Increased |
| Forkhead box protein D4-like 4 | Q8WXT5 | 70 | Increased |
| Alkyldihydroxyacetonephosphate synthase, peroxisomal | O00116 | 53 | Increased |
| Probable phosphoglycerate mutase 4 | Q8N0Y7 | 7826 | Increased |
| Mediator of RNA polymerase II transcription subunit 20 | Q9H944 | 253 | Increased |
| Erythrocyte band 7 integral membrane protein | P27105 | 62 | Increased |
| Zinc finger protein 268 | Q14587 | 4495 | Increased |
| Hydroxysteroid 11-beta-dehydrogenase 1-like protein | Q7Z5J1 | 599 | Increased |
| Zinc finger CCCH domain-containing protein 7B | Q9UGR2 | 268 | Increased |
| DNA-directed RNA polymerase III subunit RPC1 | O14802 | 54 | Increased |
| Tudor domain-containing protein 6 | O60522 | 115 | Increased |
| MKL/myocardin-like protein 2 | Q9ULH7 | 1531 | Increased |
| Adenosine 3'-phospho 5'-phosphosulfate transporter 2 | Q9H1N7 | 123 | Increased |
| Cytosolic beta-glucosidase | Q9H227 | 50 | Increased |
| Keratin, type I cuticular Ha1 | Q15323 | 239 | Increased |
| Serpin B10 | P48595 | 64 | Increased |
| Protein-glutamine gamma-glutamyltransferase Z | Q96PF1 | 64 | Increased |
| Bromodomain adjacent to zinc finger domain protein 1A | Q9NRL2 | 150 | Increased |
| Glyceraldehyde-3-phosphate dehydrogenase | P04406 | 201 | Increased |
| Ubiquitin-protein ligase E3B | Q7Z3V4 | 165 | Increased |
| Zinc finger protein 514 | Q96K75 | 67 | Increased |
| Pleckstrin homology domain-containing family O member 2 | Q8TD55 | 52 | Increased |
| Phenylalanine-4-hydroxylase | P00439 | 144 | Increased |
| Nuclear factor of activated T-cells, cytoplasmic 1 | O95644 | 60 | Increased |
| Protein shisa-6 homolog | Q6ZSJ9 | 272 | Increased |
| DNA repair protein RAD52 homolog | P43351 | 187 | Increased |
| WD repeat-containing protein 88 | Q6ZMY6 | 217 | Increased |
| IQ domain-containing protein D | Q96DY2 | 378 | Increased |
| Vacuolar protein sorting-associated protein 13B | Q7Z7G8 | 55 | Increased |
| NADH dehydrogenase (ubiquinone) complex I, assembly factor 6 | Q330K2 | 156 | Increased |
| Retinoic acid early transcript 1G protein | Q6H3X3 | 176 | Increased |
| Zinc finger protein 791 | Q3KP31 | 147 | Increased |
| Type II inositol 1,4,5-trisphosphate 5-phosphatase | P32019 | 121 | Increased |
| F-box/WD repeat-containing protein 8 | Q8N3Y1 | 142 | Increased |
| Zinc finger protein 862 | O60290 | 98 | Increased |
| Lysine-specific demethylase 2B | Q8NHM5 | 283 | Increased |
| Leucine-rich repeat-containing protein 32 | Q14392 | 216 | Increased |
| Potassium voltage-gated channel subfamily KQT member 5 | Q9NR82 | 172 | Increased |
| Serine/arginine-rich splicing factor 11 | Q05519 | 96 | Increased |
| Anoctamin-1 | Q5XXA6 | 277 | Increased |
| Protein phosphatase 1L | Q5SGD2 | 183 | Increased |
| Cingulin-like protein 1 | Q0VF96 | 130 | Increased |
| Spastin | Q9UBP0 | 160 | Increased |
| Protein CNPPD1 | Q9BV87 | 181 | Increased |
| Multifunctional protein ADE2 | P22234 | 123 | Increased |
| Death-inducer obliterator 1 | Q9BTC0 | 150 | Increased |
| T-lymphocyte surface antigen Ly-9 | Q9HBG7 | 94 | Increased |
| EF-hand calcium-binding domain-containing protein 11 | Q9BUY7 | 120 | Increased |
| Protein VPRBP | Q9Y4B6 | 103 | Increased |
| Interstitial collagenase | P03956 | 18143 | Decreased |
| Alpha-2-macroglobulin | P01023 | 4 | Decreased |
| Histone H2B type 1-M | Q99879 | 5 | Decreased |
| Antithrombin-III | P01008 | 7641 | Decreased |
| Keratin, type II cytoskeletal 1 | P04264 | 92465 | Decreased |
| Histone H3.1t | Q16695 | 12 | Decreased |
| Plasminogen activator inhibitor 1 | P05121 | 2299 | Decreased |
| Histone H2A type 1-D | P20671 | 122387 | Decreased |
| Histone H2A type 1-B/E | P04908 | 79385 | Decreased |
| Keratin, type I cytoskeletal 9 | P35527 | 675 | Decreased |
| 40S ribosomal protein SA | P08865 | 3 | Decreased |
| Complement C3 | P01024 | 4 | Decreased |
| Integrin beta-1 | P05556 | 9920 | Decreased |
| Histone H4 | P62805 | 11230 | Decreased |
| Endoplasmin | P14625 | 10062 | Decreased |
| Thyroxine-binding globulin | P05543 | 2361 | Decreased |
| 60S ribosomal protein L7a | P62424 | 1396 | Decreased |
| Triosephosphate isomerase | P60174 | 371 | Decreased |
| 72 kDa type IV collagenase | P08253 | 30 | Decreased |
| ATP synthase subunit beta, mitochondrial | P06576 | 12 | Decreased |
| Annexin A5 | P08758 | 10797 | Decreased |
| 60S ribosomal protein L7 | P18124 | 353 | Decreased |
| Myosin light chain 6B | P14649 | 14504 | Decreased |
| Peroxiredoxin-2 | P32119 | 16 | Decreased |
| Ezrin | P15311 | 53 | Decreased |
| 60S ribosomal protein L24 | P83731 | 170 | Decreased |
| Complement component C7 | P10643 | 314 | Decreased |
| Complement C4-A | P0C0L4 | 6283 | Decreased |
| Collagen alpha-3(VI) chain | P12111 | 4 | Decreased |
| Apolipoprotein M | O95445 | 3 | Decreased |
| Lactotransferrin | P02788 | 3 | Decreased |
| 60S ribosomal protein L11 | P62913 | 60 | Decreased |
| Peptidyl-prolyl cis-trans isomerase B | P23284 | 21 | Decreased |
| Protein-glutamine gamma-glutamyltransferase 2 | P21980 | 3 | Decreased |
| Myosin regulatory light chain 12A | P19105 | 648 | Decreased |
| Hemicentin-1 | Q96RW7 | 149 | Decreased |
| 40S ribosomal protein S29 | P62273 | 3 | Decreased |
| Zinc finger matrin-type protein 4 | Q9H898 | 21970 | Decreased |
| 40S ribosomal protein S25 | P62851 | 14 | Decreased |
| Protein PRR14L | Q5THK1 | 19458 | Decreased |
| 3-hydroxymethyl-3-methylglutaryl-CoA lyase, cytoplasmic | Q8TB92 | 20 | Decreased |
| Vacuolar protein sorting-associated protein 13A | Q96RL7 | 70 | Decreased |
| Ig gamma-3 chain C region | P01860 | 15 | Decreased |
| Acetyl-CoA carboxylase 1 | Q13085 | 52 | Decreased |
| Rho guanine nucleotide exchange factor 28 | Q8N1W1 | 7 | Decreased |
| Protein Daple | Q9P219 | 66 | Decreased |
| T-complex protein 1 subunit zeta | P40227 | 50 | Decreased |
| 40S ribosomal protein S15a | P62244 | 37 | Decreased |
| Perilipin-4 | Q96Q06 | 1877 | Decreased |
| DENN domain-containing protein 5A | Q6IQ26 | 25644 | Decreased |
| Olfactory receptor 5AC2 | Q9NZP5 | 826 | Decreased |
| Apolipoprotein B-100 | P04114 | 43 | Decreased |
| Coatomer subunit beta' | P35606 | 57 | Decreased |
| GTPase IMAP family member 7 | Q8NHV1 | 75 | Decreased |
| Hemopexin | P02790 | 12 | Decreased |
| Proteasome subunit beta type-6 | P28072 | 13 | Decreased |
| Ribosomal protein S6 kinase beta-2 | Q9UBS0 | 7 | Decreased |
| 40S ribosomal protein S3 | P23396 | 3 | Decreased |
| Rab effector Noc2 | Q9UNE2 | 177 | Decreased |
| Bromodomain-containing protein 4 | O60885 | 21 | Decreased |
| 10 kDa heat shock protein, mitochondrial | P61604 | 2 | Decreased |
| 40S ribosomal protein S15 | P62841 | 20 | Decreased |
| Kelch domain-containing protein 8A | Q8IYD2 | 108 | Decreased |
| Protein FAM160B2 | Q86V87 | 18 | Decreased |
| Alpha-2-antiplasmin | P08697 | 196640 | Decreased |
| Tetratricopeptide repeat protein 30B | Q8N4P2 | 48 | Decreased |
| Antigen peptide transporter 1 | Q03518 | 19 | Decreased |
| Endonuclease 8-like 1 | Q96FI4 | 27 | Decreased |
| Annexin A1 | P04083 | 2 | Decreased |
| Ig kappa chain V-I region CAR | P01596 | 33 | Decreased |
| Olfactory receptor 9K2 | Q8NGE7 | 27 | Decreased |
| Alpha-1B-glycoprotein | P04217 | 992 | Decreased |
| Solute carrier family 25 member 36 | Q96CQ1 | 2 | Decreased |
| FH1/FH2 domain-containing protein 3 | Q2V2M9 | 28 | Decreased |
| Pericentrin | O95613 | 14 | Decreased |
| Contactin-2 | Q02246 | 14 | Decreased |
| Semaphorin-4B | Q9NPR2 | 7 | Decreased |
| Zinc finger protein 583 | Q96ND8 | 14 | Decreased |
| CD48 antigen | P09326 | 9 | Decreased |
| SET and MYND domain-containing protein 5 | Q6GMV2 | 21 | Decreased |
| Myelin basic protein | P02686 | 7 | Decreased |
| TRMT1-like protein | Q7Z2T5 | 4 | Decreased |
| Diencephalon/mesencephalon homeobox protein 1 | Q8NFW5 | 36 | Decreased |
| Feline leukemia virus subgroup C receptor-related protein 2 | Q9UPI3 | 15 | Decreased |
| Pantetheinase | O95497 | 4 | Decreased |
| Phosphatidylinositide phosphatase SAC2 | Q9Y2H2 | 22 | Decreased |
| Zinc finger and BTB domain-containing protein 11 | O95625 | 386 | Decreased |
| Dual specificity mitogen-activated protein kinase kinase 1 | Q02750 | 2541 | Decreased |
| Protein CutA | O60888 | 12 | Decreased |
| Vimentin | P08670 | 19 | Decreased |
| Plexin-D1 | Q9Y4D7 | 20 | Decreased |
| Spermatogenesis-associated protein 16 | Q9BXB7 | 19 | Decreased |
| 60S ribosomal protein L15 | P61313 | 2 | Decreased |
| Collagen alpha-1(I) chain | P02452 | 7 | Decreased |
| Histone-lysine N-methyltransferase PRDM9 | Q9NQV7 | 24 | Decreased |
| Putative ankyrin repeat domain-containing protein 31 | Q8N7Z5 | 4 | Decreased |
| Adenosine deaminase domain-containing protein 1 | Q96M93 | 29 | Decreased |
| Trinucleotide repeat-containing gene 18 protein | O15417 | 21 | Decreased |
| Ubiquitin carboxyl-terminal hydrolase 24 | Q9UPU5 | 15 | Decreased |
| Leukocyte tyrosine kinase receptor | P29376 | 23 | Decreased |
| Protein SOGA3 | Q5TF21 | 14 | Decreased |
| Differentially expressed in FDCP 8 homolog | Q6ZN54 | 4 | Decreased |
| Midasin | Q9NU22 | 14 | Decreased |
| Polyribonucleotide nucleotidyltransferase 1, mitochondrial | Q8TCS8 | 2 | Decreased |
| Optineurin | Q96CV9 | 7 | Decreased |
| Isoamyl acetate-hydrolyzing esterase 1 homolog | Q2TAA2 | 15 | Decreased |
| Zinc finger protein 717 | Q9BY31 | 2 | Decreased |
| Neutral alpha-glucosidase C | Q8TET4 | 23 | Decreased |
| Meprin A subunit alpha | Q16819 | 2 | Decreased |
| SPARC | P09486 | 7 | Decreased |
| High mobility group protein HMG-I/HMG-Y | P17096 | 4 | Decreased |
| 60S ribosomal protein L12 | P30050 | 14 | Decreased |
| Transmembrane protein 132C | Q8N3T6 | 22 | Decreased |
| Endothelial protein C receptor | Q9UNN8 | 8 | Decreased |
| GAS2-like protein 2 | Q8NHY3 | 17 | Decreased |
| Tetratricopeptide repeat protein 7B | Q86TV6 | 23 | Decreased |
| Coagulation factor X | P00742 | 7 | Decreased |
| Interferon-induced protein with tetratricopeptide repeats 1B | Q5T764 | 4 | Decreased |
| Cullin-3 | Q13618 | 2 | Decreased |
| Leucine-rich repeat LGI family member 2 | Q8N0V4 | 51 | Decreased |
| F-box only protein 3 | Q9UK99 | 30 | Decreased |
| Coiled-coil domain-containing protein 168 | Q8NDH2 | 20 | Decreased |
| Dynein heavy chain domain-containing protein 1 | Q96M86 | 2 | Decreased |
| Centrosomal protein of 44 kDa | Q9C0F1 | 14 | Decreased |
| Eosinophil peroxidase | P11678 | 7 | Decreased |
| Ubinuclein-2 | Q6ZU65 | 17 | Decreased |
| Zinc finger protein 319 | Q9P2F9 | 3 | Decreased |

*accession number is the code under which the identified protein is deposited in the SwissProt database, **^†^**change of expression level is expression level of each protein in the depressive status compared to the remission status.
